# On exploiting nonparametric kernel-based probabilistic machine learning over the large compositional space of high entropy alloys for optimal nanoscale ballistics

**DOI:** 10.1038/s41598-024-62759-9

**Published:** 2024-07-22

**Authors:** K. K. Gupta, S. Barman, S. Dey, S. Naskar, T. Mukhopadhyay

**Affiliations:** 1 Amrita School of Artificial Intelligence, Amrita Vishwa Vidyapeetham, Coimbatore, India; 2 Department of Mechanical Engineering, National Institute of Technology Silchar, Silchar, India; 3 School of Engineering, University of Southampton, Southampton, UK

**Keywords:** High-velocity impact, High entropy alloys, *AlCoCrFeNi*, Machine learning assisted molecular dynamics simulation, Optimum HEAs with conflicting objectives, Nonparametric kernel-based probabilistic machine learning, Engineering, Aerospace engineering, Mechanical engineering

## Abstract

The large compositional space of high entropy alloys (HEA) often presents significant challenges in comprehensively deducing the critical influence of atomic composition on their mechanical responses. We propose an efficient nonparametric kernel-based probabilistic computational mapping to obtain the optimal composition of HEAs under ballistic conditions by exploiting the emerging capabilities of machine learning (ML) coupled with molecular-level simulations. Compared to conventional ML models, the present Gaussian approach is a Bayesian paradigm that can have several advantages, including small training datasets concerning computationally intensive simulations and the ability to provide uncertainty measurements of molecular dynamics simulations therein. The data-driven analysis reveals that a lower concentration of *Ni* with a higher concentration of *Al* leads to higher dissipation of kinetic energy and lower residual velocity, but with higher penetration depth of the projectile. To deal with such conflicting computationally intensive functional objectives, the ML-based simulation framework is further extended in conjunction with multi-objective genetic algorithm for identifying the critical elemental compositions to enhance kinetic energy dissipation with minimal penetration depth and residual velocity of the projectile simultaneously. The computational framework proposed here is generic in nature, and it can be extended to other HEAs with a range of non-aligned multi-physical property demands.

## Introduction

High entropy alloys (HEAs) are multi-principal element alloys consisting of five or more primary alloying elements with atomic percentages ranging from 5 to 35%^1,2^. The presence of a large number of principal elements in HEAs leads to mutual solubility and high configuration entropy, which promotes the formation of solid solutions with uniform phase transitions^3,4^. HEAs with simple solid solution phases outperform conventional alloys in structural and functional properties. Because of their large material compositional space and exceptional structural and functional properties, such as high hardness, ultimate tensile strength, ductility, high corrosion and wear resistance, HEAs have a high potential for a wide range of applications^5–11^. To take advantage of such properties, HEAs have been used in aerospace, sea-vessels^12–15^, high-temperature applications^16,17^, and cryogenic applications^18^. Among HEAs, the *AlCoCrFeNi* HEA has gained particular interest due to its distinctive single-phase microstructure. For example, Yang et al*.*^19^ fabricated *AlCoCrFeNi* HEAs with different *Al* concentrations and studied their mechanical and microstructural properties. It was observed that *AlCoCrFeNi* HEA exhibits a single FCC crystal structure with *Al* concentration of 0.1. In another study, Joseph et al*.*^20^ reported that *Al*_*0.3*_*CoCrFeNi* HEA exhibits remarkable work-hardening which is attributed to its single-phase (FCC) structure. Due to the evidence gathered from the past literature, *AlCoCrFeNi* HEAs are computationally modeled as a single-phase (FCC) crystal system to study its mechanical response and underlying mechanism. Such as, Jiang et al*.*^21^ conducted MD simulations to investigate the mechanical and deformation behavior of single FCC crystal *Al*_*x*_*CoCrFeNi* (*x* = 1–2, molar ratio) HEAs subjected to uniaxial tension under varying conditions. Vu et al*.*^22^ investigated the influences of grain size, temperature, and tension strain rate on mechanical properties and deformation behaviour of *Al*_*0.3*_*CoCrFeNi* HEA. Their findings revealed that elevated temperatures lead to temperature-induced softening, causing a reduction in connecting force between atoms.

A few recent studies reported promising capabilities of high entropy alloys for designing and developing materials against high-velocity impact^23–25^. For such applications, materials are subjected to high-velocity impact with high strain deformation^26^, where the strain rate is higher than ~ 10^2^ s^−1^. The microstructural transitions during high strain rate deformation of the HEA systems have been widely explored in the past. For instance, Kumar et al*.*^27^ investigated the plastic deformation behaviour of *Al*_*0.1*_*CoCrFeNi* HEA under high strain rate compression. The *Al*_*0.1*_*CoCrFeNi* HEA exhibited exceptional work-hardening capability irrespective of strain rates. Gangireddy et al*.*^28,29^ compared *Al*_*0.3*_*CoCrFeNi* (single phase FCC) with *Al*_*0.7*_*CoCrFeNi* (dual phase FCC-BCC) at a strain rate of 10^−3^ s^−1^ and 10^3^ s^−1^. The single-phase HEA system exhibited extraordinary strain rate sensitivity and work hardening capability due to low stacking fault energy (SFE). The dual-phase HEA system demonstrated higher strength due to the refined microstructure with a large number of interphase boundaries. Choudhuri et al*.*^30,31^ conducted the ballistic impact test on the *AlCoCrFeNi*_*2.1*_ HEA system and investigated the ballistic responses with microstructural deformation mechanisms. From the ballistic responses, it was observed that the *AlCoCrFeNi*_*2.1*_ HEA plates were partially penetrated, plugged, and fully penetrated with projectile velocities at 803 m/s, 1159 m/s, and 1388 m/s, respectively. Li et al*.*^32^ reported the high resistance to shear failure of the *Al*_*0.3*_*CoCrFeNi* HEA system under high-velocity deformation. Muskeri et al*.*^33^ conducted a ballistic impact test on the single-phase *Al*_*0.1*_*CoCrFeNi* HEA with high projectile velocities ranging from 500 to 1000 m/s considering the microstructural deformation behaviour. During the ballistic impact test, ductile deformation failure was observed in partial penetration, plug formation, and full penetration cases.

A few experimental investigations presented in the preceding paragraph highlight that *AlCoCrFeNi* HEA has promising capabilities against dynamic impact and high strain-rate deformations. Hence, it is essential to assess the ballistic behaviour of such a material system by considering large-scale variations in its compositional space for achieving optimal performance. However, it is challenging to perform such analysis experimentally due to the constraints associated with time, cost, and precision^34^. In contrast, performing molecular dynamics simulations have demonstrated the capability in revealing materials responses efficiently at the atomic level with adequate accuracy^35^. To mitigate these constraints, several research groups utilized molecular dynamics (MD) simulations to investigate the nanoscale ballistic performance of various prospective barrier materials^36–41^. Different HEA systems are also explored for their promising ballistic capabilities in an MD environment. For example, Tang and Li^42^ reported that *Mn* free Cantor alloy (*CrMnFeCoNi*) offers excellent resistance to failure under high-velocity impact. In another study, Sircar and Patra^43^ investigated the basis plane-specific shock response of Cantor alloy. Singh et al*.*^44^ reported the shock wave propagation in *CoCrCuFeNi* HEA as a response to an ultra-short shock pulse. Such literature indicates that the research community has started showing interest in exploring the ballistic performance of complex materials systems like HEA by utilizing MD simulations. However, characterizing the ballistic performance of HEAs as a function of large-scale variations in their compositional space remains a challenge. To address such challenges, we propose integrating machine learning (ML) approaches with MD simulations to map the entire compositional space of HEAs in a computationally efficient framework^45–52^. In this article, by integrating MD simulations with the computationally efficient nonparametric kernel-based probabilistic Gaussian process ML model, ballistic responses (such as kinetic energy dissipation (*ΔKE*), penetration depth (*δ*) and residual velocity of the projectile (*V*_*r*_)) of *AlCoCrFeNi* HEA would be mapped with the variations in the alloying composition of the individual elements. The ML based simulation framework would further be extended in conjunction with multi-objective genetic algorithm to identify the optimal HEA elemental composition for enhancing the kinetic energy dissipation with minimal penetration depth and residual velocity of the projectile.

The specific contribution of this study is the investigation of the critical ballistic performances of *AlCoCrFeNi* HEAs considering the large compositional space which is only feasible due to introduction of the machine learning-assisted efficient computational framework. It is worth noting that the functional behaviour of HEAs depends primarily on their compositional space, making it crucial to capture the entire continuous domain of variation in elemental concentration. In this context, we would computationally couple MD simulations, nonparametric kernel-based probabilistic Gaussian processes model, and the multi-objective genetic algorithm (MOGA) for extensive data-driven investigations. Hereafter, the article is organized as follows, section Modeling and simulation elaborates on the computational methodology utilized in the present investigation, section Results and discussion presents the numerical results including model validation from multiple perspectives and new results based on the efficient ML based computational framework and finally, and the following sections provide summary and concluding remarks.

## Modeling and simulation

**Figure 1:**
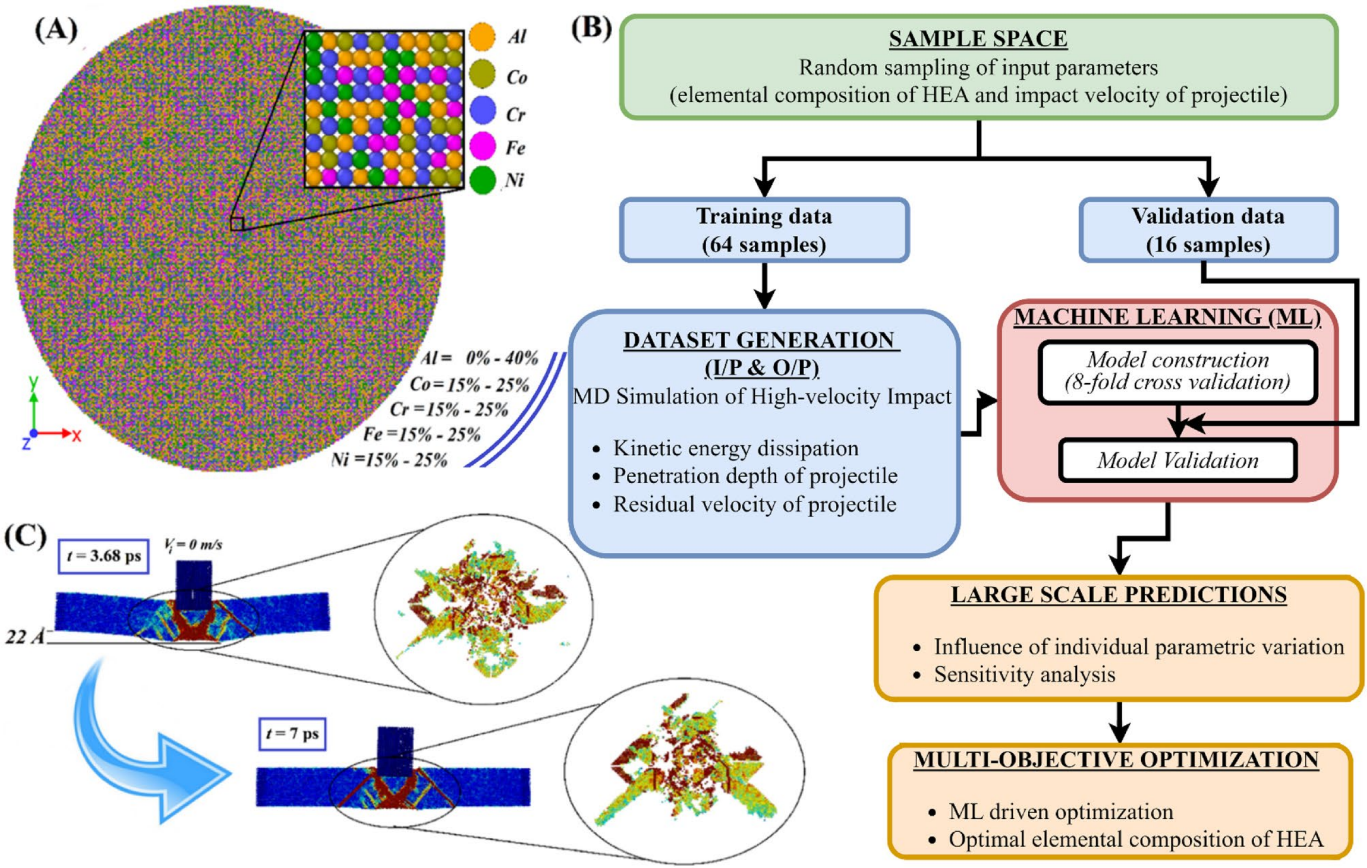
Machine learning-based computational framework coupled with molecular dynamics simulations. The seamless integration of machine learning and MD simulation is exploited here to unravel the deep insights concerning the atomic composition of *AlCoCrFeNi* HEA for achieving optimal ballistic performance. (**A**) Sobol sequence sampling-based typical random configurations of *AlCoCrFeNi* HEA modelled in LAMMPS environment. (**B**) Flow diagram of the complete computational framework adopted in the present study. (**C**) Post-impact atomistic deformation mechanism of HEA configurations suggested by large-scale physics-based investigation.

This section presents the methodology adopted to integrate the nonparametric kernel-based probabilistic Gaussian process machine learning algorithm with MD simulations to investigate the ballistic performance of *AlCoCrFeNi* HEA (refer to Fig. 1). At the first stage, the input features such as the atomic fraction of constituent elements (*Al*, *Co, Cr, Fe,* and *Ni*) of HEA and impact velocity (*V*_*i*_) of the projectile are (quasi-)randomly distributed within the parametric range of variation (considered parametric bound of the input features: *Fe* [15%, 25%], *Ni* [15%, 25%], *Co* [15%, 25%], *Cr* [15%, 25%], *Al* [0–40%], *V*_*i*_ [400 m/s, 1100 m/s]) by enforcing Sobol sequence sampling. In this manner, the sample space for training (64 samples) and validating (16 samples) the machine learning model is constructed. The random distribution of individual parameters is illustrated in Fig. S1 of the supplementary material. The conventional design of HEAs considers elemental composition of individual constituent elements in the range of 5–35%^4^. Further, the reported literature suggests that the increase in *Al* concentration in *AlCoCrFeNi* leads to the design of low-density alloy with enhanced hardness due to the inherent FCC to BCC transition on the microstructural level^19,53^. With this understanding, in the present study, the *Al* concentration is varied from 0 to 40% (highest), in accordance with the experimentally synthesized HEA configurations of *AlCoCrFeNi* reported by Gorrse et al*.*^54^, while all other constituent elements are maintained in between 15 to 25% concentrations. The *AlCoCrFeNi* HEA demonstrates great potential in surface engineering, where the objective is to enhance the hardness of the surface^55,56^. Such alloys have potential applications in the surface coating of turbine blades, aeronautical structures, and other areas where high to hypervelocity impact occurs frequently. Hence, in the present study, the impact velocity of the projectile is stochastically varied from 400 m/s (4 Å/ps) to 1100 m/s (11 Å/ps) to capture medium (10^−1^–10^2^ s^−1^), high (10^2^–10^4^ s^−1^), and dynamic strain-rate (> 10^4^ s^−1^) deformation and characterize the ballistic performance of the HEA configurations^32,57^. The MD simulations of high-velocity impact are performed on different configurations of *AlCoCrFeNi* following the (quasi-)randomly distributed input sample space. Compared to conventional machine learning models, the present Gaussian approach is a Bayesian paradigm that can have several advantages, including small training datasets concerning computationally intensive simulations and having the ability to provide uncertainty measurements of molecular dynamics simulations therein. The ability of the machine learning framework to incorporate inevitable variability in molecular dynamics simulations corresponding to each realization in the form of mean and standard deviation can address the issue of repeatability comprehensively. Unlike traditional regression models, which assume linear correlations between variables, GP models are adaptable and may account for complicated interactions between input data and output predictions. It is worth noting that, despite the unusually small sample size used here for training the ML models, the constructed Sobol sequence-based elemental composition samples capture an extensive portion of the parametric range of variation (see Fig. S1 of the supplementary material). This implies that an efficient generalized ML model for HEAs can be obtained by running a small number of computationally expensive algorithmically selected MD simulations^40,41,45–49^.

To perform the simulation of impact of the projectile in the LAMMPS^58^ environment, *AlCoCrFeNi* HEAs with randomly distributed compositional space are modelled as cylindrical (*radius* = 215 Å and *thickness* = 60.52 Å) target plates. A width of 15 Å on the target plate’s periphery is clamped by imposing zero force and zero velocity. Rigid cylindrical (*radius* = 28 Å and *height* = 78 Å) projectiles of gold are utilized to perform the atomistic simulations of impact. The interaction among the constituent elements of *AlCoCrFeNi* HEA is modeled by utilizing the *EAM/alloy* force-field^59^. The same force field is used to model the monolithic gold nano-projectile. The interface between constituent elements of HEA and the projectile (*Au*) is modeled by using the *Lennard–Jones* (*L–J*) force-field^60^, wherein the calculation of the *L–J* parameters is done following Tang and Li^42^. The *L–J* parameters utilized in the simulations are presented in Table 1. An iteration time-step of 0.1 fs is utilized to perform the MD simulations. The potential energy of the simulation setup is minimized for 2.5 ps before beginning the MD simulations of high-velocity impact. By imposing the NPT ensemble for 10 ps, the pressure and temperature of the energy-minimised simulation setup are equilibrated at 0 bar and 300 K, respectively. Further, the projectile is impacted on the target plate within the *NVE* ensemble. The instantaneous variation in the kinetic energy of the projectile is assessed to evaluate the kinetic energy dissipated (*ΔKE*) by the target plate, which can be calculated as

1 $$\Delta KE = KE_{i} - KE_{f}$$

**Table 1:** *L–J* parameters used to model the interaction between HEA configurations and Au nano-projectile.

Atom pair	ε (eV)	σ (Å)
Al–Au	70.62	3.47
Co–Au	11.76	2.74
Cr–Au	12.17	2.81
Fe–Au	11.33	2.76
Ni–Au	12.17	2.72

where *KE*_*i*_ refers to the initial kinetic energy of the projectile and *KE*_*f*_ refers to the post-impact kinetic energy of the projectile. Also, the temporal variation in the projectile’s velocity is observed to obtain the post-impact residual velocity (*V*_*r*_). The extent of deflection/damage (penetration depth (*δ*)) of the target plate is evaluated by observing the temporal variation in the transverse displacement of the projectile. The dataset generated by quasi-random sampling-based MD simulation is utilized to train and validate the Gaussian Process-based machine learning model. It is to be noted that the constructed machine learning model is validated by a separate set of samples which are not utilized while training the model. It ensures that the constructed models are capable of accurate generalization without any prediction bias. A detailed description of the Gaussian Process machine learning model is provided in section SM1 of the supplementary material. The constructed Gaussian Process models utilize *isotropic Matern 2.5* kernel with a kernel scale of 5.5 and basis function ‘*zero*’. Based on the proposed framework we develop a nonparametric kernel-based probabilistic computational mapping between the fraction of constituent elements of *AlCoCrFeNi* HEA and impact velocity of projectiles with kinetic energy dissipated by HEA plate, depth of penetration and residual velocity of projectiles.

The Gaussian Process models are further exploited to perform genetic algorithm based multi-objective optimization, wherein, the optimal elemental compositions of *AlCoCrFeNi* HEA are proposed to maximize the kinetic energy dissipation of HEA with minimum possible penetration depth and residual velocity. A detailed description of the genetic algorithm based multi-objective optimization algorithm is provided in section SM2 of the supplementary material. The optimization is performed by developing a MATLAB code with a population size of ‘100’, a cross-over rate of ‘0.8’ and by enforcing the adaptive mutation function. It is to be noted that the proposed optimization framework employs a computationally efficient ML model as the fitness function (not just the initial 80 samples). This enables the optimization algorithm to search across the entire parametric range of variation, utilizing the ML model's ability to rapidly evaluate the fitness of potential solutions. Here the population size of 100 refers to the genetic algorithm's search space over the continuous domain of parametric variation, rather than being constrained by the initial 80 samples utilized for model creation. Thus, we are able to exploit the generalization capability of the ML model over the entire design space for genetic algorithm. While performing the optimization to find the optimal solution to the multi-objective problem, the atomic fractions of the constituent elements are restricted in the same range as mentioned before in this section.

## Results and discussion

This section presents the numerical results obtained from the coupled machine learning (ML) driven MD simulations framework. To validate the MD simulations based ballistic analyses, the specific kinetic energy dissipation (ΔKE^*^) of an aluminum plate is compared with the published findings^37^. The specific kinetic energy dissipation (ΔKE^*^) is the kinetic energy dissipated by the target with respect to the mass of the impact zone. The structural configurations of the cylindrical target aluminum plate (effective radius: 200 Å and thickness: 24.3 Å) and cylindrical gold projectile (diameter: 77 Å and height: 81 Å) are maintained as reported in Dewapriya and Miller^37^. The current MD simulations result in the ΔKE^*^ of aluminum as 3.03 MJ/kg, 3.69 MJ/kg, and 4.67 MJ/kg at the impact velocity of 500 m/s, 750 m/s, and 1000 m/s, respectively. These observations are found to be in good agreement with the values reported by Dewapriya and Miller^37^, i.e. 3.12 MJ/kg at 500 m/s, 3.98 MJ/kg at 750 m/s, and 4.67 MJ/kg at 1000 m/s. The validatory simulations establish the reliability of the computational approach used in the MD simulation of a high-velocity impact. With sufficient confidence in the simulations of high-velocity impact, further extensive MD simulations are performed on different configurations of *AlCoCrFeNi* HEA to construct the training and validation dataset. The detailed process of sample preparation and data generation is explained in section “Modeling and simulation”.

**Figure 2:**
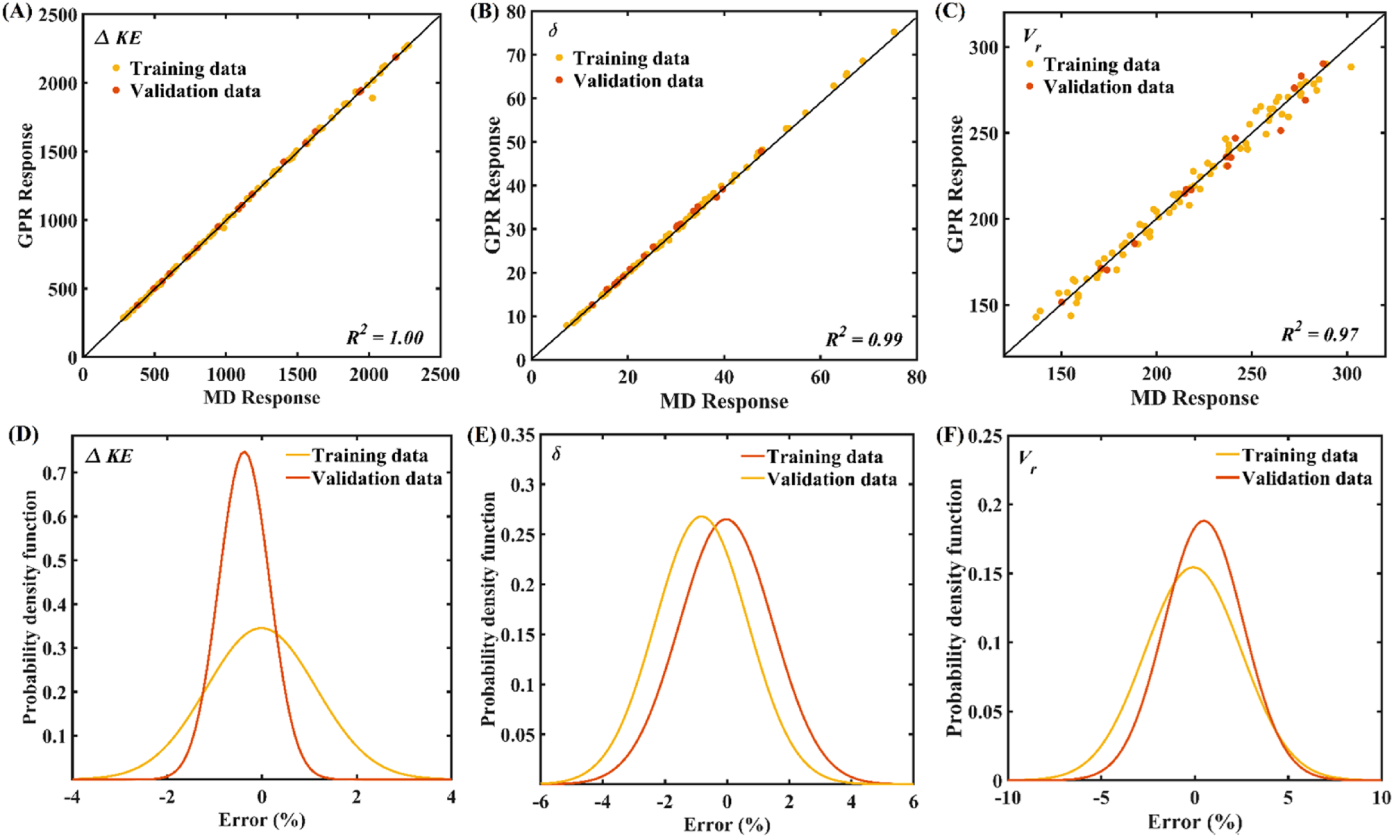
Validation of Gaussian process-based ML model. (**A**) Scatter plot between MD simulations derived *ΔKE* and Gaussian process predicted *ΔKE*. (**B**) Scatter plot between MD simulations derived *δ* and Gaussian Process predicted *δ*. (**C**) Scatter plot between MD simulations derived *V*_*r*_ and Gaussian Process predicted *V*_*r*_. (**D**) Probability density function plots of percentage error in the prediction of *ΔKE*. (**E**) Probability density function plots of percentage error in the prediction of *δ*. (**F**) Probability density function plots of percentage error in the prediction of *V*_*r*_.

The sample space constructed by performing MD simulations for (quasi-)randomly sampled input parameters is utilized to develop the Gaussian process based ML model. In the present study, an individual ML model is constructed for the considered quantities of interest, i.e*.* kinetic energy dissipation (*ΔKE*), penetration depth (*δ*), and residual velocity (*V*_*r*_). The validation of developed ML models is presented in Fig. 2. Figure 2A–C illustrates the scatter plots between the responses obtained from the MD simulation and predicted by the Gaussian Process model for *ΔKE, δ,* and *V*_*r*,_ respectively. It can be noticed from these scatter plots that the true and predicted responses are significantly closer to the linear diagonal line, which indicates an accurate ML model construction. The corresponding percentage error in the predictions is illustrated in Fig. 2D–F in terms of probability density function (pdf) plots. It is evident from the error plots that the distribution of percentage error in predictions remains in the range of ± 10% for each response, while more instances show less error. Note that the training and validation sizes for obtaining the results in Fig. 2 are based on the aforementioned number of simulations. With adequate confidence established in the computational efficiency of constructed Gaussian Process models, the models are further utilized to perform large-scale predictions.

**Figure 3:**
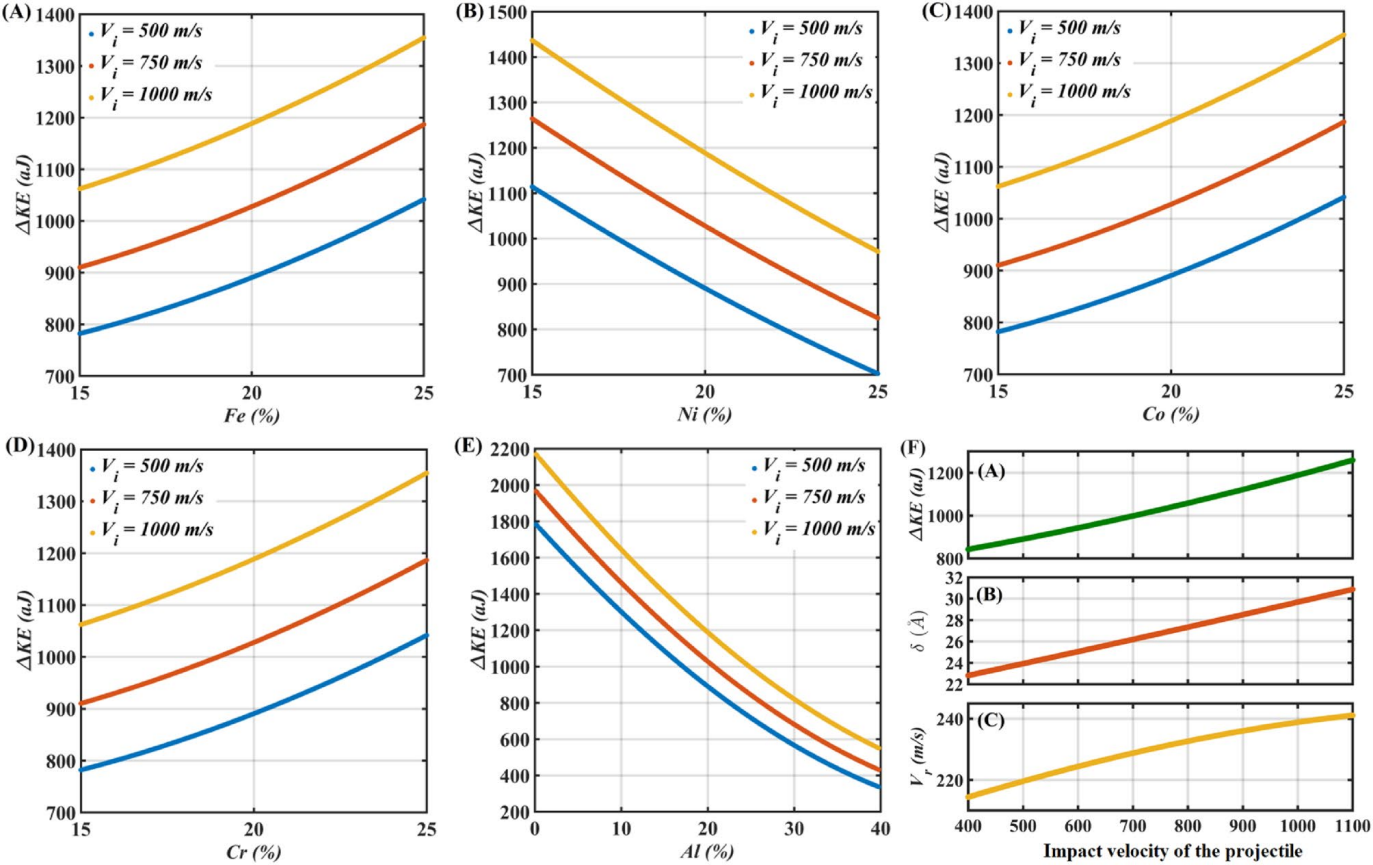
General trends of ballistic parameters. (**A**–**E**) Variation in kinetic energy dissipation (*ΔKE*) with atomistic composition at different impact velocities. (**F**) Variation in ballistic parameters *ΔKE*, *δ* and *V*_*r*_ as a function of impact velocity ranging from 400 to 1100 m/s.

The input parameters are randomly distributed within the parametric range by using Monte Carlo sampling (~ 10^4^ samples). At first, the influence of increasing impact velocity on the responses is explored, wherein the impact velocity is stochastically varied in the range of 400–1100 m/s, while the equiatomic fractions (each 20%) of constituent elements (*Al, Co, Cr, Fe* and *Ni*) of HEA are maintained. The impact velocity-dependent ballistic performance of *AlCoCrFeNi* HEA is depicted in Fig. 3F. It can be noticed from the figure that with the increase in impact velocity all three responses (*ΔKE, δ,* and *V*_*r*_) increase simultaneously, which indicates a positive correlation of impact velocity with the ballistic responses of HEA. The individual ballistic responses are then evaluated in the context of variation in the atomic fraction of each constituent element of HEA at three different impact velocities (500 m/s, 750 m/s, and 1000 m/s). The atomic fraction of *Fe*, *Ni*, *Co,* and *Cr* is stochastically varied from 15 to 25% and the atomic fraction of *Al* is stochastically varied from 0 to 40%. At a time, the concentration of only one element is varied within the corresponding parametric range and the remaining elements are maintained with equiatomic fractions. The individual atomic fractions dependent kinetic energy dissipation (*ΔKE*) of HEA is presented in Fig. 3A–E. The results highlight that the variation in *ΔKE* as a function of the atomic fraction of an individual element demonstrates the same trend regardless of the impact velocity of the projectile. Also, an increase in the atomic fractions of *Fe*, *Co*, and *Cr* indicates the subsequent increase in the kinetic energy dissipation of the HEA (see Fig. 3A–D), whereas, an increase in atomic fractions of *Ni* and *Al* results in a decrease in the kinetic energy dissipation of the HEA (see Fig. 3B,E).

Similar observations can be inferred from the individual atomic fractions dependent penetration depth (*δ*) which is illustrated in Fig. S2 of the supplementary material. The observation in terms of *ΔKE* and *δ* reveals that with the increase in penetration depth the kinetic energy dissipation increases and vice-versa. This can be explained by the increase in plastic deformation of the target plate with the increase in penetration depth. Unlike the influence of atomic concentrations of constituent elements on the kinetic energy dissipation (*ΔKE*) and depth of penetration (*δ*) of HEA, the residual velocity (*V*_*r*_) does not exhibit a sharp increase or decrease, except for the case of an increase in *Al* concentrations (see Fig. S3 of the supplementary material). Note that the overall slopes of the curves presented in Fig. 3, Figs. S2 and S3, provide an indication (/preliminary understanding) of the corresponding relative importance of the compositional elements to the response quantities. It is further revealed from the figures that the increase in *Ni* and *Al* concentration has a negative influence on the kinetic energy dissipation and penetration depth of HEA, whereas, an increase in *Al* concentration exhibits a negative influence on the projectile’s residual velocity. The remaining elements show a positive correlation to the corresponding response parameters. It is to be noted that the superior ballistic performance of the material must exhibit high kinetic energy dissipation with reduced residual velocity and penetration depth of the projectile.

**Figure 4:**
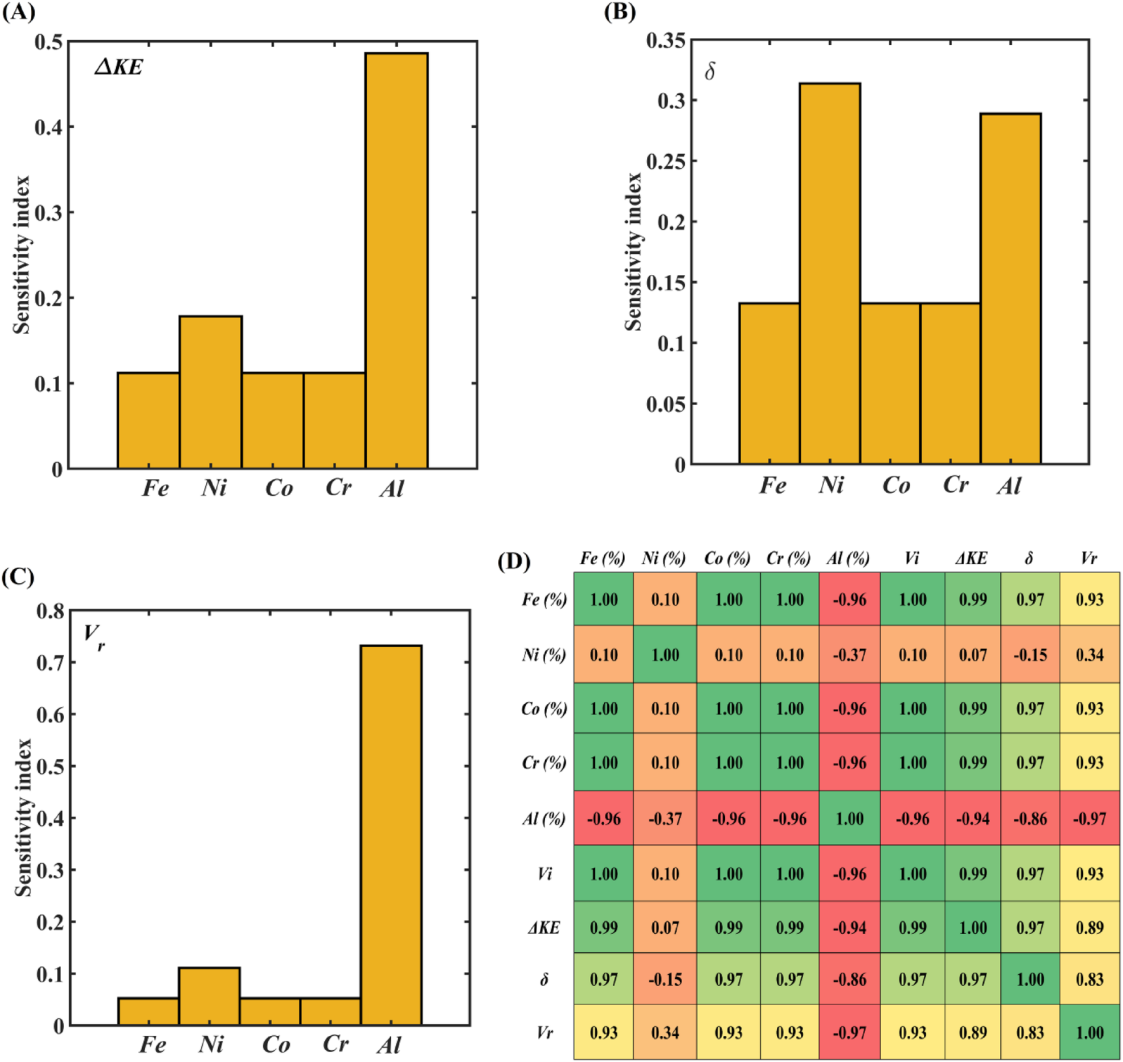
Statistical insights of individual input parameters on the ballistic performance of *AlCoCrFeNi* HEA. Data-driven sensitivity analysis of constituent elements on the (**A**) kinetic energy dissipation (*ΔKE*) of *AlCoCrFeNi*, (**B**) projectile’s depth of penetration (*δ*), (**C**) projectile’s residual velocity (*V*_*r*_). Note that the sensitivity analysis results only provide absolute values, and they cannot show the negative or positive correlations between input and output parameters. Thus, a separate correlation analysis is further carried out. (**D**) Correlogram to show the correlations among input features and ballistic responses.

The atomic composition-based computational mapping developed through Gaussian Process predictions is utilized to perform a data-driven sensitivity analysis. In this regard, the relative coefficient of variation is evaluated for the cases of individual variation in atomic composition. The data-driven sensitivity analysis is performed for all three responses and presented in Fig. 4A–C. The impact velocity is kept constant at 750 m/s during the sensitivity analysis because the responses show the same pattern of variation regardless of the impact velocity. The sensitivity analysis presented in Fig. 4A–C demonstrates that irrespective of the responses, variations in atomic fractions of *Fe*, *Co*, and *Cr* have a similar statistical significance on the ballistic performance of *AlCoCrFeNi* HEA. In contrast, the compositional variation in *Ni* and *Al* concentration shows a significant difference in the parametric correlations with the ballistic measures (*ΔKE*, *δ*, and *V*_*r*_) of the HEA. The correlation mapping of input features with the ballistic responses is presented in Fig. 4D, which indicates a strong negative correlation of *Al* concentration on the ballistic performance measures. Apart from the *Al* concentration, the *Ni* concentration also exhibits a mild negative influence on the ballistic responses. All the other input features (compositional change in *Fe*, *Co*, and *Cr* concentration) including the impact velocity exhibit a strong positive correlation with the ballistic responses. Note here that the correlation coefficient varies from -1 (red color) to 1 (green color) where -1 indicates strong negative correlation of an individual parameter and 1 indicates strong positive correlation. Subsequently, any values in between the range of − 1 to 1 give a quantification of the degree of positive or negative correlation. It is worth mentioning that the results presented in Fig. 4 are in good agreement with the preceding discussions based on Fig. 3, Figs. S2 and S3.

**Figure 5:**
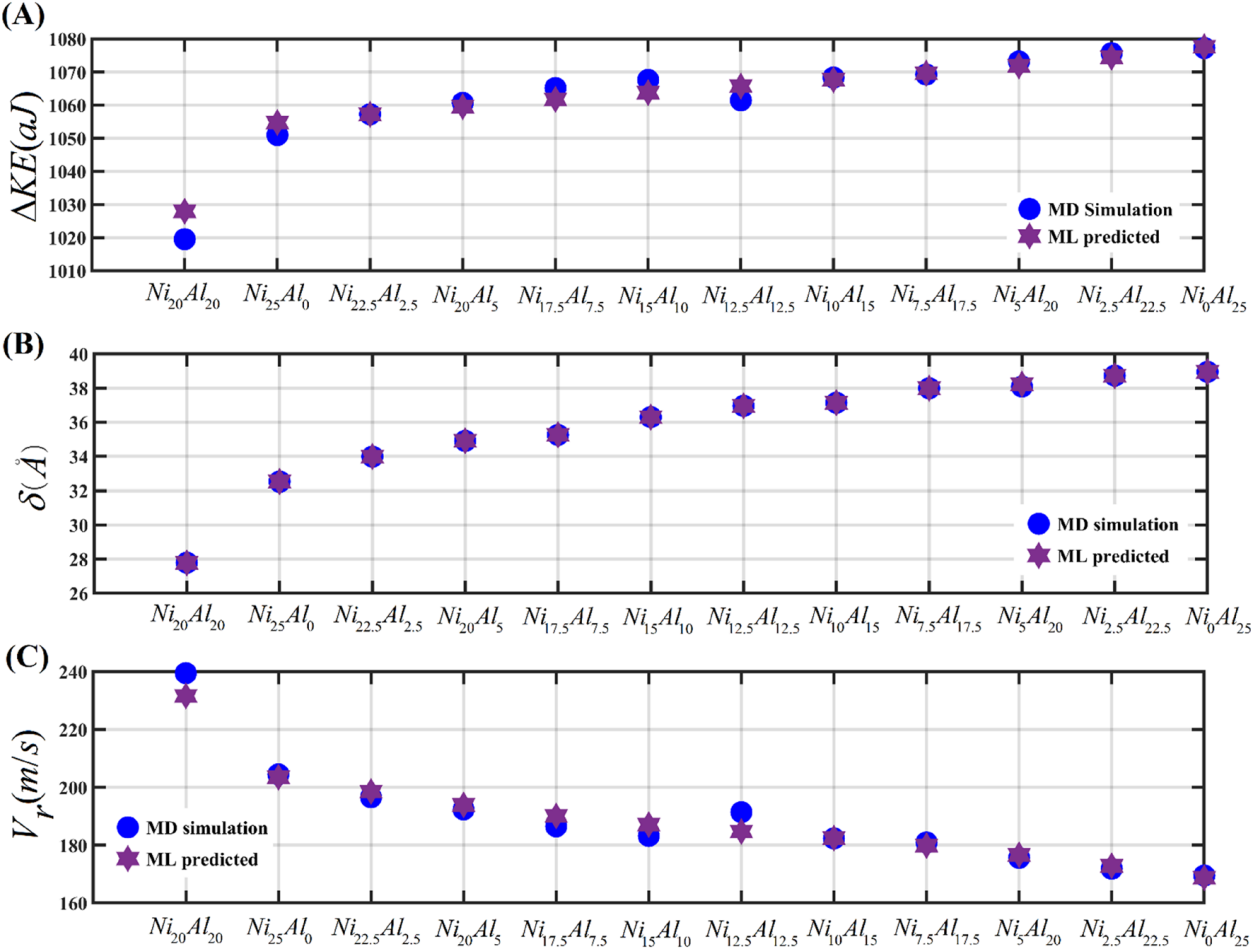
Combined influence of *Ni* and *Al* concentration on the ballistic performance of *AlCoCrFeNi* HEA. (**A**) kinetic energy dissipation (*ΔKE*), (**B**) projectile’s depth of penetration (*δ*), (**C**) projectile’s residual velocity (*V*_*r*_).

With the above understanding, in the next step, the combined composition of *Fe*, *Co*, and *Cr* is maintained at 75%, while the atomic fractions of *Ni* and *Al* are varied so that the collective atomic fraction of *Ni* and *Al* remains at 25%. The combined variation in the atomic fractions of *Ni* and *Al* is accomplished by decreasing the atomic fraction of *Ni* from 25 to 0% and simultaneously increasing the atomic fraction of *Al* from 0 to 25%, with an interval of 2.5%. The ballistic performance of these HEA configurations is compared with the equiatomic *AlFeCoCrNi* HEA (see Fig. S4 of the supplementary material and Fig. 5). It is observed that the high velocity (*V*_*i*_ ≈ 750 m/s) impact of projectiles on the equiatomic *AlFeCoCrNi* HEA results in the least depth of penetration (see Fig. S4B and Fig. 5B). However, it also leads to comparatively higher post-impact kinetic energy and velocity of the projectile (see Fig. S4A,C), indicating low kinetic energy dissipation offered by equiatomic *AlFeCoCrNi* HEA. The increase in *Al* concentration and a simultaneous decrease in *Ni* concentration increases the transverse displacement of the projectile, which is evident in Fig. S4B and Fig. 5B. The highest transverse depth traveled by the projectile is observed for the HEA configuration with 25% *Al* and 0% *Ni*. The increase in transverse displacement of the projectile is a consequence of increased plastic deformation of the target plate with the increase in *Al* concentration. The increased penetration depth of the projectile offered by the HEA configuration with 0% *Ni* and 25% *Al* exhibits relatively higher kinetic energy dissipation (see Fig. 5A) and the least residual velocity of the projectile (see Fig. 5C). Such observations establish that with the increase in atomic fraction of *Al* the ease in penetration of the projectile increases which also enhances the deflection of the target and with increased plastic deformation the kinetic energy dissipation increases.

**Figure 6:**
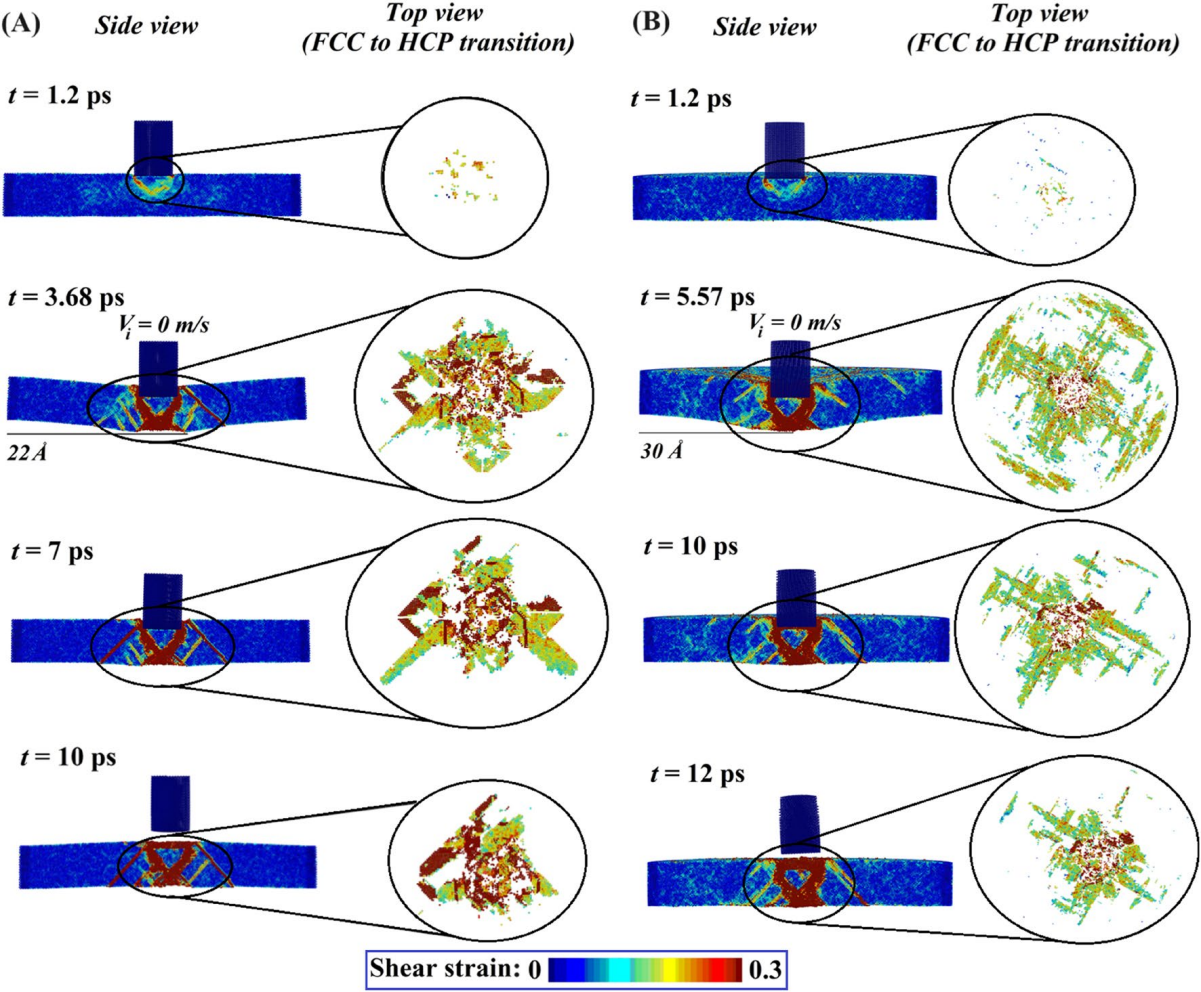
Temporal post-impact structural evolution of *AlCoCrFeNi* HEAs. The frame by frame atomistic trajectory of a cylindrical projectile at different time instants is shown. The evolution of shear strain bands in the target plate is also demonstrated for each individual time frame. (**A**) Structural evolution of equiatomic *AlCoCrFeNi* HEA. The contact happens with target equiatomic *AlCoCrFeNi* HEA at 1.2 ps. At 3.68 ps the projectile comes to rest with the maximum deflection observed in the rear face of target as 22 Å. At this instant, nearly 1% of the HEA atoms (localized near the impact zone) are transitioned from FCC to HCP crystal structure. At 7 ps the resisting stress-wave (in the HEA target) dependent rebound of the projectile happens. At 10 ps the projectile completely comes loose from the target and rebounds with a residual velocity. (**B**) Structural evolution of *Al*_*25*_(*FeCrCo*)_*75*_*Ni*_*0*_ HEA. The contact with target *Al*_*25*_(*FeCrCo*)_*75*_*Ni*_*0*_ HEA happens at 1.2 ps. At 5.57 ps the projectile comes to rest with the maximum deflection observed in the rear face of target as 30 Å. At this instant, nearly 3.73% of the HEA atoms (localized near the impact zone) transitioned from FCC to HCP crystal structure. At 10 ps the resisting stress-wave (in the HEA target) dependent rebound of the projectile happens. At 12 ps the projectile completely comes loose from the target and rebounds with a residual velocity.

To understand the deformation mechanics more clearly the structural evolution of equiatomic *AlCoCrFeNi* HEA (refer to Fig. 6A) as a result of high-velocity impact is compared with the *Al*_*25*_(*FeCrCo*)_*75*_*Ni*_*0*_ HEA (refer to Fig. 6B). The results reveal that the nano-projectile comes to rest earlier in the case of equiatomic HEA (at 3.68 ps) when compared with the *Al*_*25*_(*FeCrCo*)_*75*_*Ni*_*0*_ HEA (at 5.56 ps). This is explained by the higher plastic deformation observed in the case of *Al*_*25*_(*FeCrCo*)_*75*_*Ni*_*0*_ HEA. The highest deflection in the target plates is observed at the instance when the projectile comes to rest. At the instance of highest deflection in both cases, a transition from FCC lattice to HCP lattice of the particles (structural evolution) is recorded. The FCC to HCP transition is represented by roughly 1% (7763 atoms) of all atoms in equiatomic HEA. In the case of *Al*_*25*_(*FeCrCo*)_*75*_*Ni*_*0*_ HEA, however, this percentage climbs to 3.73% (29,979 atoms). It is also worth noting that in the case of equiatomic HEA, the FCC to HCP transition of atoms is restricted primarily to the vicinity of the impact zone, while for *Al*_*25*_(*FeCrCo*)_*75*_*Ni*_*0*_ HEA, the HCP atoms can be seen spreading up to the boundary region.

**Figure 7:**
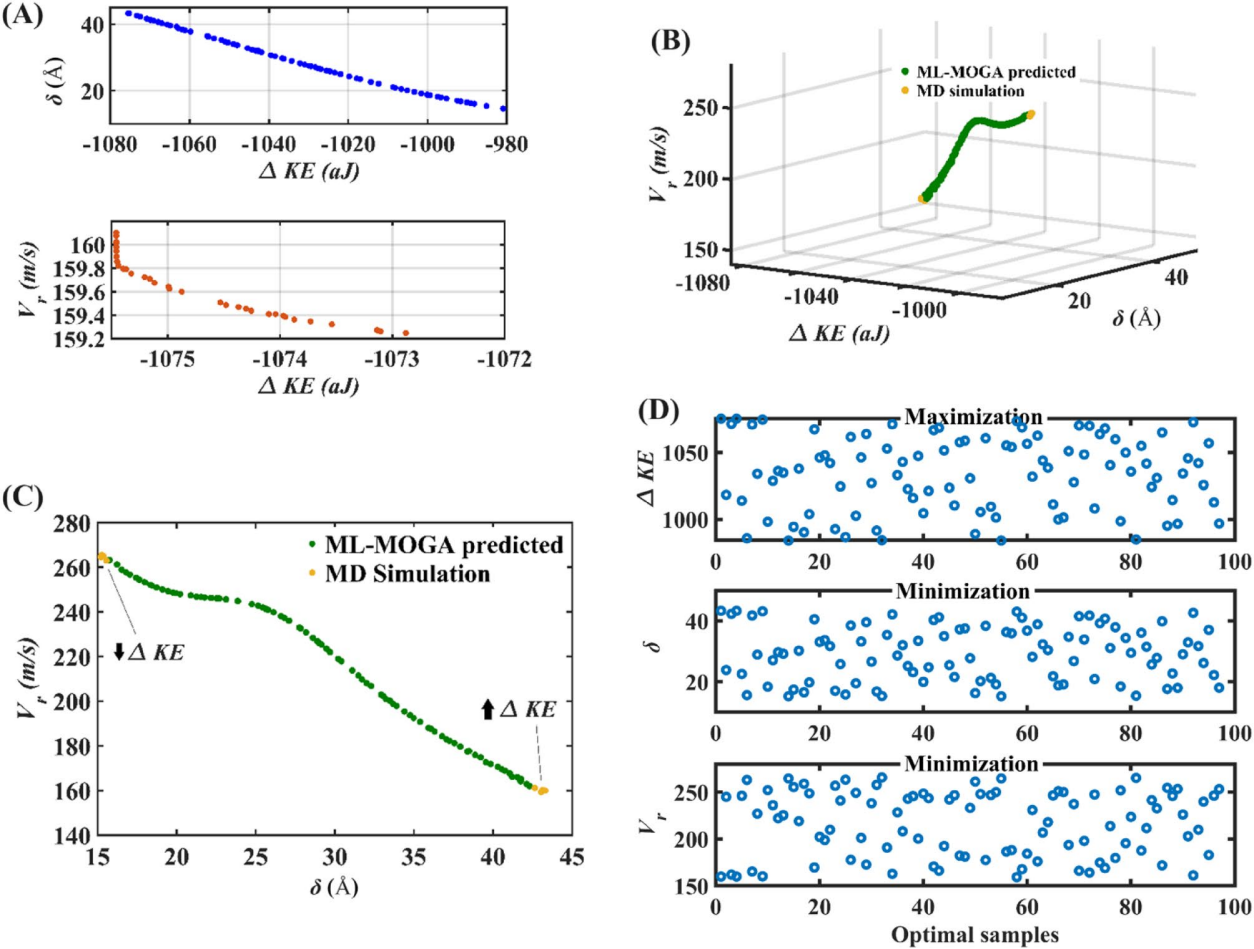
Pareto front of non-dominated solutions in terms of maximized *ΔKE* with minimized *δ* and *V*_*r*_. With the help of the presented data-driven analysis, a set of optimal compositions of *AlCoCrFeNi* HEA is suggested (refer to Tables S1–S4 of the supplementary material) for enhancing the post-impact kinetic energy dissipation with minimal residual velocity and penetration depth of the projectile. (**A**) Solutions for maximizing post-impact kinetic energy dissipation with individual minimization of residual velocity and penetration depth of the projectile. (**B**,**C**) Solutions for maximizing post-impact kinetic energy dissipation with simultaneous minimization of residual velocity and penetration depth of the projectile. The scatter points in yellow color (refer to (**B**) and (**C**)) correspond to the ballistic responses gathered by performing the MD simulations. These points denote the higher and lower kinetic energy dissipation observed as illustrated in (**C**) (↑ denotes the points with maximum observed *ΔKE* and ↓ denotes the points with minimum observed *ΔKE*). The corresponding compositional space of the *AlCoCrFeNi* HEA can be referred from Table S4 of the supplementary material. (**D**) The spread of optimal solution based on ML-MOGA optimization for maximizing ΔKE, and minimizing δ and *V*_*r*_.

With the understanding gathered in the preceding paragraphs, it is clear that increasing the *Al* concentration with simultaneously decreasing the *Ni* concentration increases kinetic energy dissipation, and also results in a decrease in the projectile’s residual velocity. Although an increase in kinetic energy dissipation with a decrease in residual velocity is a desirable phenomenon, it occurs at the expense of an increase in penetration depth (see Fig. S4B), which is undesirable in the context of barrier materials. To overcome this lacuna, in the following stage, the Gaussian Process models (for *ΔKE*,* δ* and *V*_*r*_) are employed to perform multi-objective optimization. An evolutionary algorithm is utilized to find a set of optimal atomic fractions of constituent elements of *AlCoCrFeNi* HEA to maximize the kinetic energy dissipation with minimized penetration depth and residual velocity of the projectile (see section SM4 of the supplementary material). The Pareto solution obtained from the genetic algorithm-based multi-objective optimization is illustrated in Fig. 7. The non-dominated solutions presented in Fig. 7A refer to the optimal elemental composition of *AlCoCrFeNi* HEA (as presented in Tables S1 and S2 of the supplementary material) corresponding to maximizing the post-impact kinetic energy dissipation, along with individual minimization of penetration depth and residual velocity, respectively. At first, only two responses (out of three) are considered in performing the multi-objective optimization at a time, wherein for each instant maximizing the kinetic energy dissipation is considered as a common objective. The solutions obtained from such investigation provide insight into the optimal atomic composition of *AlCoCrFeNi* HEA which could result in maximum post-impact kinetic energy dissipation with either minimum penetration depth or minimum residual velocity of the projectile. In the next step, while performing the multi-objective optimization, all three responses are considered with the intended objectives (maximizing: *ΔKE*, minimizing:* δ* and *V*_*r*_) at the same time. The Pareto solution for the same is illustrated in Fig. 7B,C, and the corresponding optimal atomic compositions of *AlCoCrFeNi* HEA are presented in Table S3 of the supplementary material. The spread of optimal solution based on ML-MOGA optimization for maximizing *ΔKE*, and minimizing *δ* and *V*_*r*_ is illustrated in Fig. 7D. For validating the optimal results, we have performed direct MD simulations further, and the outcomes are in perfect agreement with the results of ML-MOGA framework, as depicted in Fig. 7B,C. This provides additional confidence in the exploitation of machine learning over the large compositional space of AlCoCrFeNi high entropy alloys for unraveling optimal nanoscale ballistic performances. Note here that the Pareto optimal results provide a significant extent of design flexibility in terms of the compositional components for achieving the target responses as per application-specific demands.

## Summary and perspective

The large compositional space of high entropy alloys presents a significant challenge in identifying the function-specific optimal fractions of each of the constituent elements. Such analysis requires an intensive data-driven investigation, which is not always viable based on experimental methods or computationally expensive direct simulation methods (such as molecular dynamics (MD) simulation). This creates a strong rationale to develop alternative high-fidelity methods which can accurately perform data-driven investigations, while incurring relatively much lower costs compared to conventional approaches. This article proposes an efficient computational framework to obtain the optimal composition of high entropy alloys under ballistic conditions. We have proposed to exploit the emerging capabilities of machine learning through random sampling-based molecular dynamics (MD) simulation which is further integrated with Gaussian process driven multi-objective genetic algorithm. Based on the proposed framework we have developed an efficient computational mapping between the fraction of constituent elements (*Al, Co, Cr, Fe* and *Ni*) of HEA and impact velocity (*V*_*i*_) of the projectile with kinetic energy dissipated (*ΔKE*) by HEA plate, depth of penetration (*δ*) and residual velocity (*V*_*r*_) of the projectile. Compared to conventional machine learning models, the present Gaussian approach is a Bayesian paradigm that has several advantages, including computational efficiency in training the compositional space of high entropy alloys and having the ability to provide uncertainty measurements of molecular dynamics simulations therein. The ability of the machine learning framework to incorporate inevitable variability in molecular dynamics simulations corresponding to each realization in the form of mean and standard deviation can address the issue of repeatability comprehensively.

The influencing features such as the atomic fraction of constituent elements (*Fe, Ni, Co, Cr,* and *Al*) of HEA and impact velocity (*V*_*i*_) of the projectile are algorithmically sampled within the parametric bound of variation based on quasi-random Sobol sequence. Subsequently, the sample space for training and validating the machine learning model is constructed by performing respective MD simulations for high-velocity impact. The ballistic performances of the HEA in terms of kinetic energy dissipation, residual velocity and penetration depth of the projectile are recorded based on MD simulations. The computational accuracy of the constructed ML model is ascertained on the basis of percentage error in the prediction, which remains within 10% for the converged training sample size. Note that the current analysis also involves another stage of validation concerning the baseline molecular dynamics simulation framework for high-velocity impact. The Gaussian process-based machine learning model is coupled subsequently with genetic algorithm at the following stage for identifying the optimal compositions in HEA with multiple conflicting objectives. For validating the optimal results, we have performed direct MD simulations further, and the outcomes are found to be in perfect agreement with the results of ML-MOGA framework. A three-fold validation adopted here provides adequate confidence in the exploitation of machine learning over the large compositional space of AlCoCrFeNi HEAs for unraveling optimal nanoscale ballistic performances. The salient outcomes derived from the data-driven analysis presented in this article are summarized below.

1. The constructed Gaussian Process models demonstrate adequate generalization capabilities, wherein the prediction error ranges below 10% for each ML model concerning ballistic responses.
2. The impact velocity has a strong positive correlation with all three ballistic responses under consideration. With the increase in impact velocity from 400 to 1100 m/s the *ΔKE*, *δ*, and *V*_*r*_ exhibits 49.4%, 35.3%, and 12.46% increase, respectively.
3. The atomic fraction-dependent kinetic energy dissipation and depth of penetration exhibit similar trends, regardless of the impact velocity. An increasing trend in kinetic energy dissipation and depth of penetration is noticed as the atomic fractions of *Fe*, *Co*, and *Cr* are increased. In contrast, when the atomic fractions of *Ni* and *Al* increase, the kinetic energy dissipation and depth of penetration decrease.
4. A strong positive correlation between *ΔKE* and *δ* is observed (with the increase in depth of penetration the kinetic energy dissipation increases). This can be explained by the increase in plastic deformation of the target plate with the increase in penetration depth which further increases the kinetic energy dissipation.
5. An increase in *Al* concentration exhibits a sharp decline in the residual velocity of the projectile.
6. The data-driven sensitivity analysis demonstrates that regardless of the responses, variations in atomic fractions of *Fe*, *Co*, and *Cr* have a similar statistical significance on the ballistic performance of *AlCoCrFeNi* HEA. In contrast, the compositional variation in *Ni* and *Al* concentration shows a significant difference in the parametric correlations with the ballistic measures (*ΔKE*, *δ*, and *V*_*r*_) of the HEA.
7. An increase in *Al* concentration and a simultaneous decrease in *Ni* concentration increases transverse displacement of the projectile when compared with equiatomic *AlCoCrFeNi* HEA. The highest transverse depth traveled by the projectile is observed for the HEA configuration *Al*_*25*_(*CoCrFe*)_*75*_*Ni*_*0*_. The increase in transverse displacement of the projectile is a consequence of increased plastic deformation of the target plate with the increase in *Al* concentration. The increased penetration depth of the projectile offered by the HEA configuration with 0% *Ni* and 25% *Al* also results in relatively higher kinetic energy dissipation and the least residual velocity of projectile.
8. The atomistic deformation mechanics reveals that, at the instance of highest deflection, a transition from FCC lattice to HCP lattice of the particles (structural evolution) takes place, wherein the percentage of atoms participating in the process is significantly higher in *Al*_*25*_(*FeCrCo*)_*75*_*Ni*_*0*_ HEA compared to the equiatomic configurations. This leads to a substantially higher energy dissipation for *Al*_*25*_(*FeCrCo*)_*75*_*Ni*_*0*_ HEA.
9. The computationally efficient Gaussian Process models are further exploited to perform multi-objective genetic algorithm based optimization, which can provide Pareto solutions and the corresponding optimal points for designing the *AlCoCrFeNi* HEA having enhanced kinetic energy dissipation with reduced depth of penetration and residual velocity of the projectile.

## Conclusions

We have proposed a generic error-inclusive Bayesian computational approach for HEAs by exploiting machine learning over the large compositional space for unraveling optimal nanoscale ballistic performance with conflicting objectives. More interestingly, Pareto optimal solutions are obtained, leading to a wide range of design flexibilities for multi-objective application-specific functionalities. The proposed machine learning-based approach can be readily extended to different other HEAs and various multi-physical properties with conflicting objectives and inherent simulation variabilities.

### Supplementary Information

Supplementary Information 1.

Supplementary Information 2.

## Data Availability

The datasets used and/or analysed during the current study are available from the corresponding author on reasonable request.
