# Supplementary material for: On exploiting nonparametric kernel-based probabilistic machine learning over the large compositional space of high entropy alloys for optimal nanoscale ballistics
Source: Sci Rep. 2024 Jul 22;14:16795. doi: 10.1038/s41598-024-62759-9 (PMC11263686; doi:10.1038/s41598-024-62759-9)
Supplement: Supplementary file 2 — Supplementary Information 2. [file 41598_2024_62759_MOESM2_ESM.pdf]

Supplementary Material

On exploiting nonparametric kernel-based probabilistic machine learning over the large compositional space of high entropy alloys for optimal nanoscale ballistics

K. K. Gupta[a], S. Barman[b], S. Dey[b*], S. Naskar[c], T. Mukhopadhyay[c*]

[a]*Amrita School of Artificial Intelligence, Coimbatore, Amrita Vishwa Vidyapeetham, India*
[b]*Department of Mechanical Engineering, National Institute of Technology Silchar, India*
[c]*School of Engineering, University of Southampton, Southampton, UK*

**Email address: sudip@mech.nits.ac.in (SD), t.mukhopadhyay@soton.ac.uk (TM)*

In this supplementary document, we have provided the mathematical details concerning two critical aspects of the current investigation: nonparametric kernel-based probabilistic machine learning and multi-objective genetic algorithm. Supporting additional numerical results are furnished here in Figures S1 to S4. The optimal compositional fractions associated with the Pareto solutions shown in Figure 7 of the main paper are also presented here in tabulated form (refer to Table S1, S2 and S3 along with the validation results of Table S4).

Contents

SM1. Nonparametric kernel-based probabilistic machine learning algorithm 1

SM2. Multi-Objective genetic algorithm for conflicting targets 3

SM3. Additional numerical results 4

SM4. Optimal Solutions 8

SM1. Nonparametric kernel-based probabilistic machine learning algorithm

A nonparametric kernel-based probabilistic machine learning such as Gaussian process (GP) is a Bayesian modeling technique that doesn't provide a specific form to the relationship between the input variables and the target. Based on the available data, GP identifies the relationship between input variables and target variables. This method has advantages over many ML algorithms due to its integration of several ML tasks i.e., estimation of hyperparameters, model training, and uncertainty estimation. GP aims to relate the observed responses to an arbitrary regression function $g(x)$ with an additive normally distributed noise (ξ). This is expressed by the following equation [1]:

$$y = g(x) + \xi \tag{1}$$

where y refers to the response values and x refers to the predictor variables. A Gaussian process $g(x)$ can be specified by $g(x) \sim GP\left(m(x), k(x, x')\right)$, wherein $m(x)$ and $k(x, x')$ denote the mean and covariance functions as given by the following equations

$$m(x) = E\left[g(x)\right] \tag{2}$$

$$k(x, x') = E\left[\left(g(x) - m(x)\right)\left(g(x') - m(x')\right)\right] \tag{3}$$

where

$$\begin{gathered} y \sim N\left(m(x), K(X, X) + \sigma^2 I\right), \\ K_{i,j} = k\left(x_i, x_j\right) \end{gathered} \tag{4}$$

Based on the nature of the noise (ξ) and the marginalization property of GPs, the joint prior distribution of the training output y at X and test outputs g_* at test points X_* is displayed in Eq. (5)

$$\begin{bmatrix} y \\ g_* \end{bmatrix} \sim N\left(\begin{matrix} m(X) \\ m(X_*) \end{matrix}, \begin{bmatrix} K(X, X) + \sigma^2 I & K(X, X_*) \\ K(X_*, X) & K(X_*, X_*) \end{bmatrix}\right) \tag{5}$$

As y follows the Gaussian distribution and the joint prior distribution is obtained using Eq (5), the posterior distribution of g_* can be obtained by the Eq. (6)

where,

$$\begin{gathered} p\left(y_* \mid X, y, X_*\right) \sim N\left(\bar{y}_*, \operatorname{var}(y_*)\right), \\ \bar{y}_* = m(X_*) + k(X_*, X)\left[k(X, X) + \sigma_n{}^2 I\right]^{-1}\left(y - m(X)\right) \\ \operatorname{var}(y_*) = k(X_*, X_*)\left[k(X, X) + \sigma_n{}^2 I\right]^{-1} k(X, X_*) \end{gathered} \tag{6}$$

SM2. Multi-Objective genetic algorithm for conflicting targets

As the name suggests, multi-objective optimization involves optimizing a number of objectives simultaneously. The problem becomes challenging when the objectives are of conflict to each other (like in the present case), that is, the optimal solution of an objective function is different from that of the other. In solving such problems, with or without the presence of constraints, these problems give rise to a set of trade-off optimal solutions, popularly known as Pareto-optimal solutions.

A multi-objective optimization problem involves several objective functions which are to be either minimized or maximized. As in a single-objective optimization problem, the multi-objective optimization problem may contain a number of constraints that any feasible solution (including all optimal solutions) must satisfy. Since objectives can be either minimized or maximized, we state the multi-objective optimization problem in its general form [2]:

$$\begin{aligned} &\textit{Minimize / Maximize} \quad f_m(x), \qquad m = 1,2,........,M; \\ &\textit{subject to} \quad g_j(x) \geq 0, \quad j = 1,2,.........,J; \\ &\qquad h_k(x) = 0, \quad k = 1,2,.........,K; \\ &\qquad x_i^{(L)} \leq x_i \leq x_i^{(U)}, \quad i = 1,2,....,n. \end{aligned} \tag{7}$$

A solution $x \in R^n$ is a vector of n decision variables: $x = (x_1, x_2, . . . , x_n)^T$. The solutions satisfying the constraints and variable bounds constitute a feasible decision variable space $S \subset R^n$. One of the striking differences between single-objective and multi-objective optimization is that in multi-objective optimization the objective functions constitute a multi-dimensional space, in addition to the usual decision variable space. This additional M-dimensional space is called the objective space, $Z \subset R^M$. For each solution x in the decision variable space, there exists a point $z \in R^M$ in the objective space, denoted by $f(x) = z = (z_1, z_2, . . . , z_M)^T$. To make the descriptions clear, we refer a 'solution' as a variable vector and a 'point' as the corresponding objective vector. The optimal solutions in multi-objective optimization can be defined from a mathematical concept of partial ordering. In the parlance of multi-objective optimization, the term domination is used for this purpose. In this section, the unconstrained (without any equality, inequality or bound constraints) optimization problem is discussed. The domination between two solutions is defined as follows:

Definition: A solution $x^{(1)}$ is said to dominate the other solution $x^{(2)}$, if both the following conditions are true:

1. The solution $x^{(1)}$ is no worse than $x^{(2)}$ in all objectives. Thus, the solutions are compared based on their objective function values (or location of the corresponding points ($z^{(1)}$ and $z^{(2)}$) on the objective space).

2. The solution $x^{(1)}$ is strictly better than $x^{(2)}$ in at least one objective.

SM3. Additional numerical results

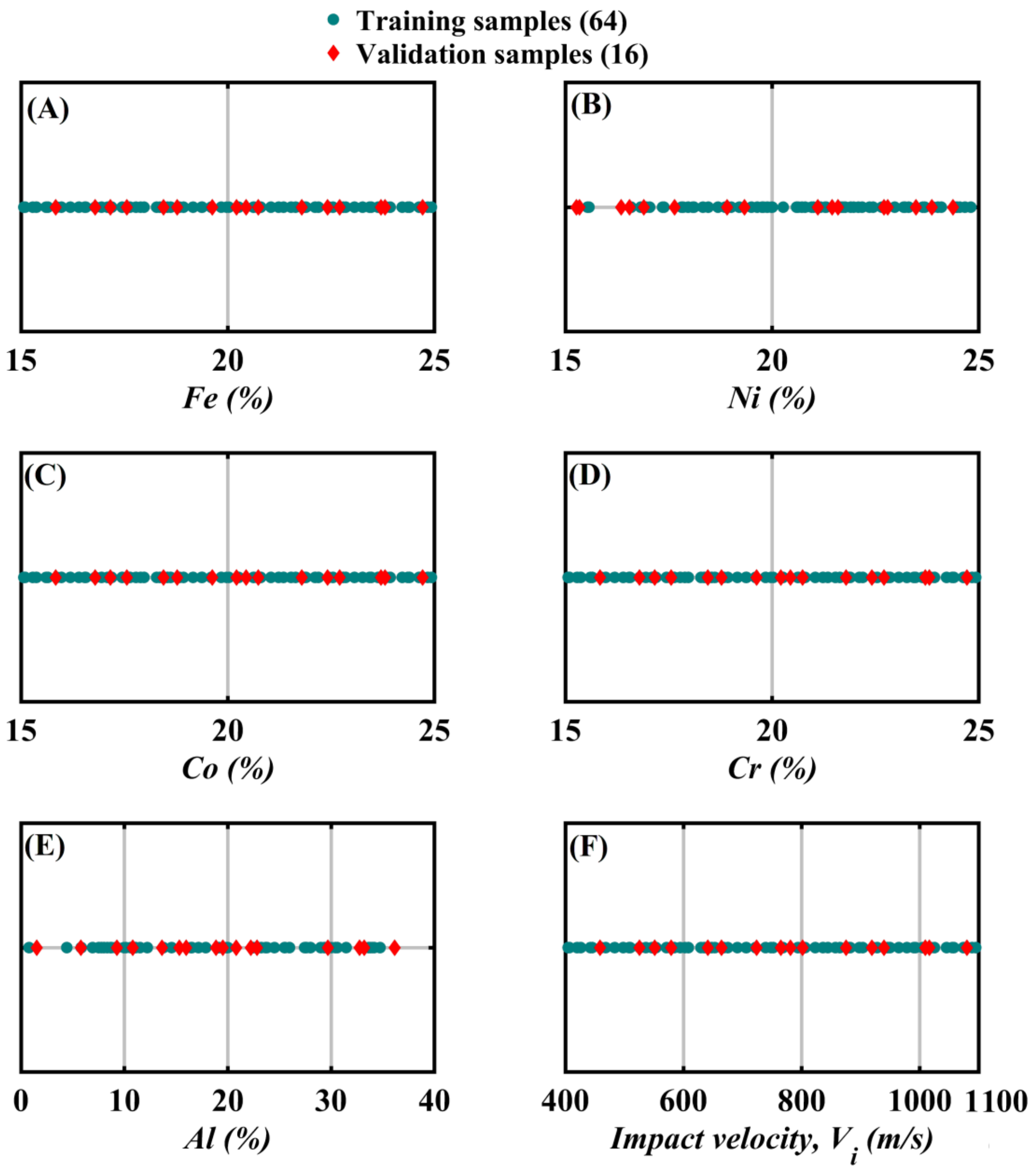

Figure S1. Random distribution of input features within the parametric bound of variation. The quasi-random Sobol sequence sampling is used to construct a sample space for forming the machine learning models.

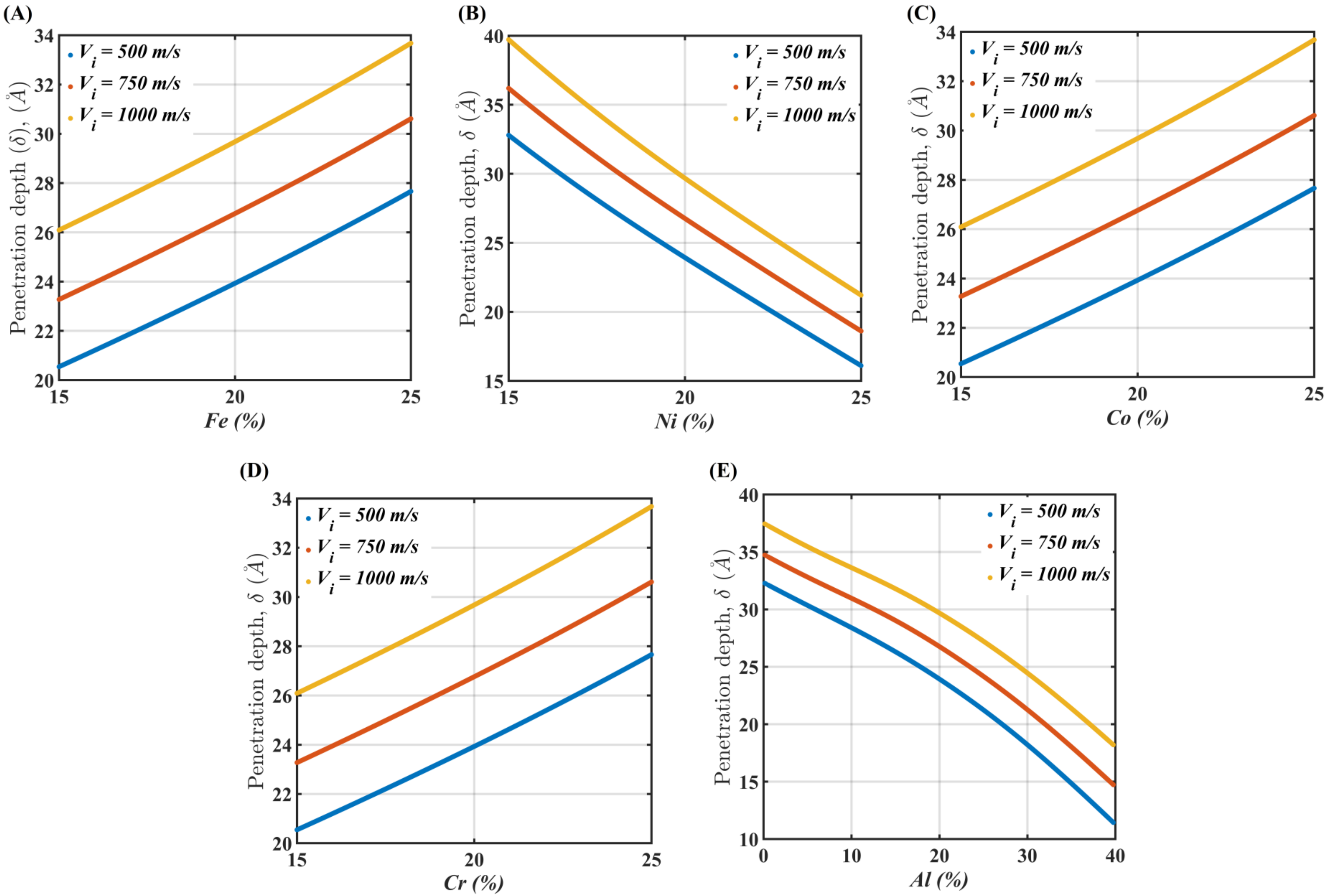

Figure S2. Variation in penetration depth (δ) with atomistic composition. Penetration depth (δ) of the projectile as a function of the variation in alloying composition of **(A)** *Fe* **(B)** *Ni* **(C)** *Co* **(D)** *Cr* and **(E)** *Al* at different impact velocities.

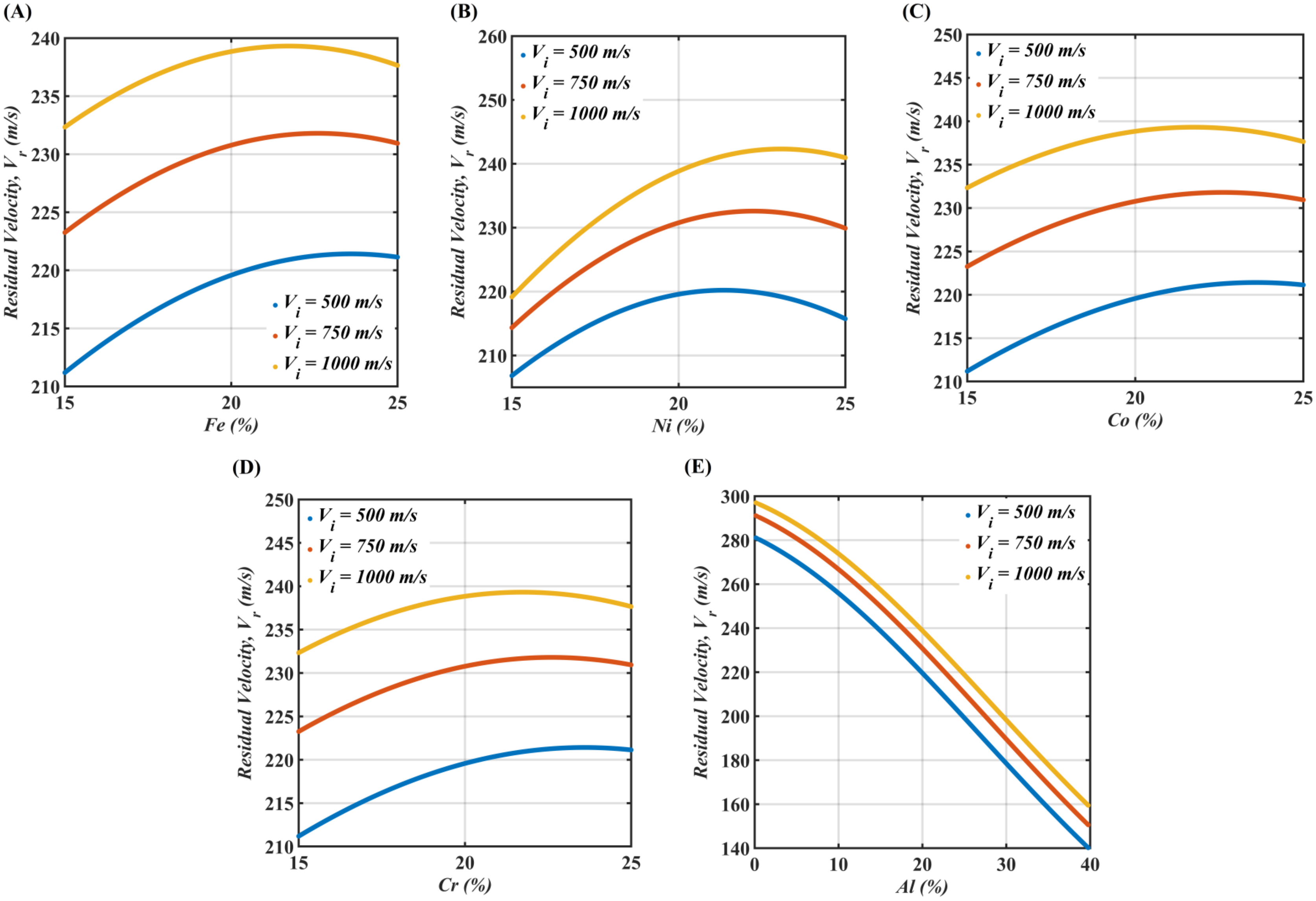

Figure S3. Variation in residual velocity (V_r) with atomistic composition. Residual velocity (V_r) with respect to the variations in alloying composition of **(A)** *Fe* **(B)** *Ni* **(C)** *Co* **(D)** *Cr* and **(E)** *Al* at different impact velocities.

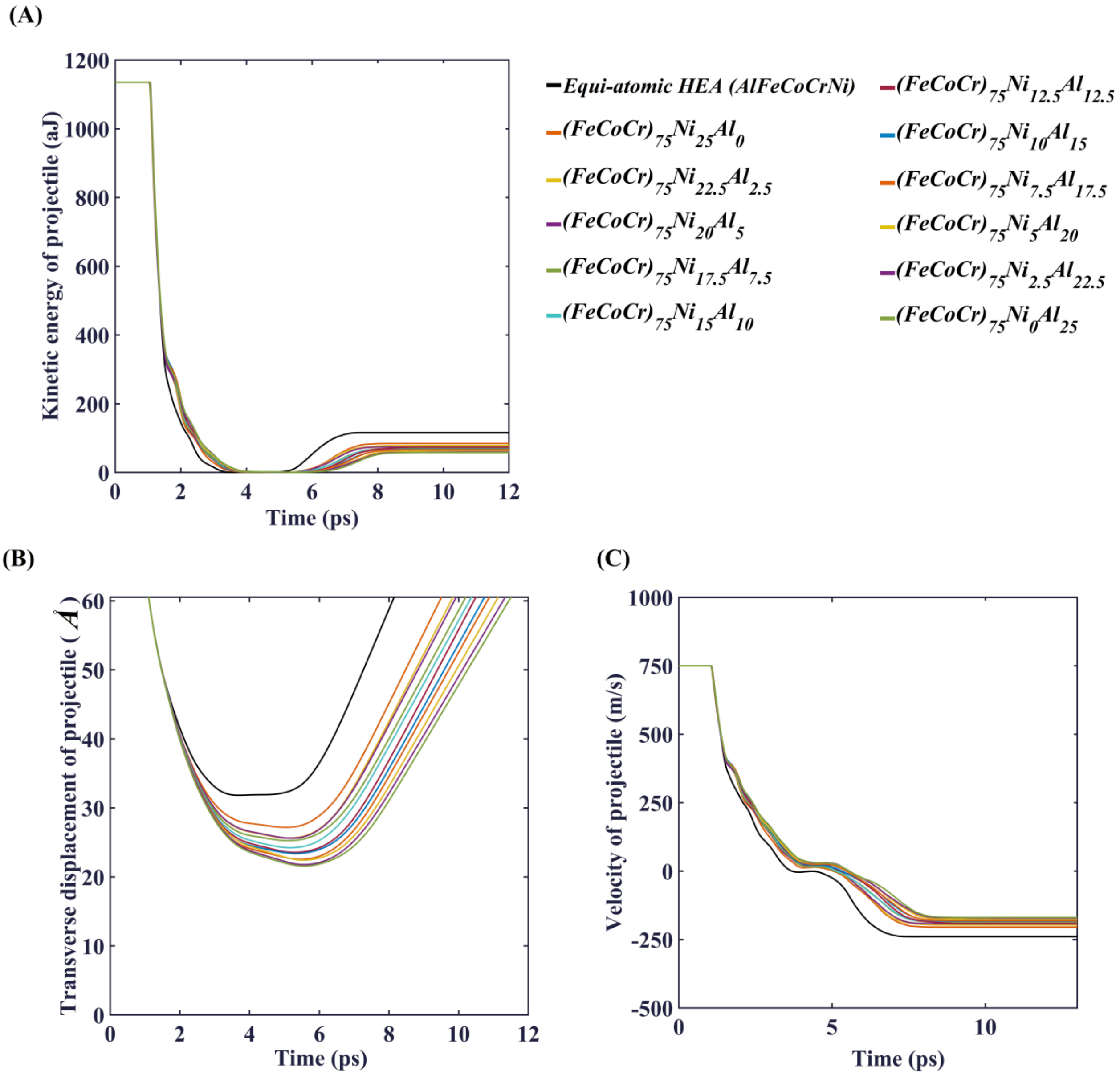

Figure S4. Temporal variation in the ballistic responses of *AlCoCrFeNi* HEA as a function of *Ni* and *Al* concentration. Comparison of temporal variation in **(A)** kinetic energy of projectile **(B)** transverse displacement of projectile **(C)** velocity of projectile for different configurations of *AlFeCoCrNi* HEA with combined variations in *Ni* and *Al* atomic fractions.

SM4. Optimal solutions

Table S1. Optimal elemental composition obtained by MOGA for maximizing the kinetic energy dissipation and minimizing the penetration depth of projectiles at the impact velocity of 750 m/s.

Fe	*Ni*	*Co*	*Cr*	*Al*	*ΔKE*	*δ*
15.74244	15.00043	16.15703	17.10108	36	-1075.45	43.32066
15.84062	15.01669	16.25471	16.94753	35.94141	-1075.3	43.26497
16.27946	15.42409	16.64995	16.36771	35.27975	-1073.29	42.60402
16.18529	15.31466	16.48822	17.13549	34.87729	-1072.44	42.26285
16.71319	15.49768	16.79729	16.71845	34.27432	-1070.81	41.68923
17.38174	15.13884	16.31215	17.44452	33.72366	-1069.86	41.25185
16.51122	15.67624	16.79035	17.38804	33.63506	-1069.1	41.08819
16.64604	15.90683	16.84821	17.3626	33.23721	-1067.9	40.69297
17.58332	15.75971	16.91416	16.93865	32.80505	-1067.02	40.32399
16.94163	16.17262	17.03655	17.44921	32.40086	-1065.63	39.89659
17.73424	15.98387	16.728	17.54762	32.00712	-1064.88	39.57125
17.64739	17.3264	16.61814	16.49366	31.91524	-1063.26	39.26774
17.81963	15.98629	17.04467	17.99147	31.15878	-1062.85	38.8072
18.05482	16.00221	17.08404	18.1175	30.74226	-1061.84	38.42935
18.13155	16.12492	17.11891	18.10127	30.52417	-1061.19	38.21313
18.0467	16.61854	17.60036	17.59025	30.14494	-1059.77	37.7905
18.24782	18.02932	17.43048	17.30546	28.98769	-1055.54	36.51771
18.60162	17.08097	17.92073	17.93908	28.45834	-1055.26	36.19938
18.33286	17.32268	18.22174	18.15062	27.97284	-1053.86	35.72524
18.58043	18.36467	17.74756	17.66626	27.64181	-1051.98	35.2556
18.69318	17.81348	18.27642	18.18371	27.0339	-1051.11	34.80438
18.76286	17.75094	18.40922	18.38181	26.69587	-1050.37	34.51348
19.02157	18.1172	18.26974	18.31368	26.27849	-1048.99	34.08089
19.09482	18.01	18.58719	18.45004	25.85859	-1048.1	33.72553
18.93107	17.99092	18.50182	18.77043	25.80645	-1048	33.68338
19.0753	18.34556	18.94965	18.77397	24.85617	-1045.37	32.78324
18.87912	18.36105	18.90462	19.45517	24.40069	-1044.27	32.38113
19.44837	18.38348	19.11317	19.08514	23.97047	-1043.23	31.9984
19.33501	19.20658	18.73874	18.63249	24.08781	-1042.65	31.96313
19.17181	18.81927	19.53927	18.87964	23.59062	-1041.87	31.59295
19.84265	18.53275	19.28628	19.87288	22.46603	-1039.5	30.66519
19.85508	19.19881	19.35311	19.27881	22.31477	-1038.45	30.42198
20.00494	19.26819	19.62951	19.49199	21.60591	-1036.7	29.80139
19.79455	19.21047	19.85221	19.755	21.38834	-1036.24	29.62576
20.133	19.41098	19.99027	19.8333	20.633	-1034.25	28.95039
20.3498	19.62278	20.11712	20.06174	19.84907	-1032.18	28.25635
20.57281	19.89375	20.0642	20.0136	19.45614	-1030.97	27.88331
20.54593	19.9896	20.1738	20.30964	18.98152	-1029.75	27.47533
20.30079	20.03515	20.32648	20.49173	18.84635	-1029.38	27.35714
20.39207	20.72446	20.12086	20.06834	18.69472	-1028.31	27.11578
20.79207	20.12446	20.52086	20.46834	18.09472	-1027.52	26.72785
20.72281	20.26845	20.43718	20.66764	17.90438	-1026.92	26.5503
20.87611	20.32269	20.77812	20.703	17.32051	-1025.49	26.07204

20.76293	20.68129	20.64038	20.80056	17.11529	-1024.64	25.84885
20.9604	20.79999	20.81761	20.78279	16.63965	-1023.39	25.45226
21.03402	20.97212	20.96072	20.92889	16.10467	-1021.96	25.00522
21.34876	21.04298	21.27465	21.13646	15.19753	-1019.76	24.2956
21.35308	21.35692	21.30955	21.45579	14.52504	-1017.86	23.737
21.51484	21.54658	21.31031	21.38913	14.23951	-1016.99	23.49196
21.75817	21.58197	21.8468	21.70765	13.10576	-1014.3	22.65819
21.92459	22.04939	21.81332	21.73155	12.48148	-1012.35	22.13511
22.11511	22.15835	22.34334	22.42327	10.96022	-1008.69	21.07066
22.41326	22.10865	22.4388	22.33311	10.70646	-1008.15	20.90719
22.48629	22.50383	22.40644	22.36291	10.2408	-1006.66	20.533
22.47379	22.49133	22.45644	22.35041	10.2283	-1006.64	20.52679
22.61459	22.75166	22.4972	22.43446	9.702319	-1005.14	20.14103
22.76143	22.96901	22.67151	22.57348	9.0248	-1003.34	19.6729
22.92938	23.03402	22.91388	22.81365	8.309282	-1001.61	19.21705
23.10126	23.11012	23.08784	23.0056	7.695378	-1000.1	18.83136
23.1396	23.23098	23.14654	23.0274	7.455671	-999.42	18.6678
23.30828	23.44421	23.33579	23.21764	6.694247	-997.43	18.18518
23.39304	23.53843	23.39168	23.27472	6.402276	-996.654	18.00121
23.35328	23.42459	23.9317	23.54673	5.743853	-995.244	17.64853
23.69753	23.87146	23.71801	23.96616	4.746961	-992.466	17.02893
24.41316	23.86282	23.56486	23.77149	4.387777	-991.641	16.8336
23.99226	24.22498	24.00525	23.89781	3.879786	-990.087	16.50931
24.15397	24.15227	24.14976	24.09108	3.452997	-989.173	16.30081
24.05192	24.3285	24.10798	24.3928	3.118882	-988.216	16.10514
24.4825	24.53148	24.57293	24.52694	1.886193	-985.147	15.46159
24.88515	24.91924	24.99769	24.94651	0.251418	-980.956	14.63785
24.88735	24.92135	25	24.94879	0.24251	-980.933	14.6335
24.88735	24.92135	25	24.94879	0.24251	-980.933	14.6335

Table S2. Optimal elemental composition obtained by MOGA for maximizing the kinetic energy dissipation and minimizing the residual velocity of projectiles at the impact velocity of 750 m/s.

Fe	*Ni*	*Co*	*Cr*	*Al*	*ΔKE*	V_r
15.00046	15.00001	15.00975	18.98978	36	-1075.46	160.1023
15.0005	17.47698	15.00235	16.52018	36	-1072.88	159.2507
15.36804	15.5581	15.74722	17.32685	36	-1074.87	159.6013
15.28945	16.15316	15.53232	17.02522	36	-1074.26	159.4385
15.13614	15.00035	15.61946	18.24421	36	-1075.46	159.8993
15.01865	17.26027	15.05263	16.66847	36	-1073.11	159.2647
15.42036	15.45072	15.83788	17.29128	36	-1074.99	159.6268
15.05	15.00013	15.22079	18.72913	36	-1075.46	160.024
15.10696	15.00026	15.33077	18.5621	36	-1075.46	159.9797
15.07252	16.66105	15.24023	17.02628	36	-1073.73	159.3476
15.18651	16.5202	15.44131	16.85209	36	-1073.88	159.3633
15.32795	15.24089	15.80021	17.63117	36	-1075.2	159.7229
15.09991	15.00026	15.45665	18.4433	36	-1075.46	159.9467

15.28783	15.01564	15.84068	17.85609	36	-1075.44	159.821
15.35486	15.12542	15.89976	17.62018	36	-1075.32	159.7542
15.22177	16.04502	15.56223	17.17113	36	-1074.37	159.4714
15.2996	15.08171	15.82881	17.79012	36	-1075.37	159.7913
15.44453	15.93652	15.58445	17.03466	36	-1074.48	159.4872
15.11355	16.36284	15.33759	17.18614	36	-1074.04	159.4096
15.29558	15.44434	15.81444	17.44585	36	-1074.99	159.6439
15.26277	15.88841	15.64897	17.20001	36	-1074.53	159.5087
15.02766	16.8447	15.07945	17.04822	36	-1073.54	159.3228
15.3048	15.2853	15.79085	17.61927	36	-1075.16	159.7088
15.05632	15.00015	15.25063	18.69297	36	-1075.46	160.0137
15.46456	15.32415	15.79132	17.42021	36	-1075.12	159.675
15.01606	15.00005	15.078	18.90591	36	-1075.46	160.0763
15.16394	16.30403	15.48344	17.04871	36	-1074.1	159.4104
15.10323	16.42855	15.30191	17.16639	36	-1073.97	159.3961
15.13269	16.44488	15.34588	17.07665	36	-1073.95	159.3862
15.02693	17.23171	15.21446	16.52693	36	-1073.14	159.2737
15.2636	16.10845	15.47294	17.15517	36	-1074.3	159.4567
15.31233	15.05577	15.86228	17.76986	36	-1075.4	159.7957
15.28599	15.00574	15.67699	18.03147	36	-1075.45	159.8571
15.00046	15.00001	15.00975	18.98978	36	-1075.46	160.1023
15.0005	17.47698	15.00235	16.52018	36	-1072.88	159.2507

Table S3. Optimal elemental composition obtained by MOGA for maximizing the kinetic energy dissipation with simultaneously minimizing the penetration depth and residual velocity of projectiles at the impact velocity of 750 m/s.

Fe	***Ni***	***Co***	***Cr***	***Al***	ΔKE	δ	V_r
16.90007	15.08533	16.10413	15.91047	36	-1075.36	43.30087	159.962
16.90007	15.08533	16.10413	15.91047	36	-1075.36	43.30087	159.962
16.61331	15.42387	16.0857	16.01528	35.86184	-1074.68	43.12512	160.2349
15.03718	16.8445	15.78988	16.32844	36	-1073.54	43.02711	159.3592
15.81871	16.36196	16.03464	16.3057	35.479	-1072.79	42.63558	161.2098
15.27333	17.00582	15.99959	16.50504	35.21623	-1071.5	42.29756	162.0165
15.65754	16.76204	16.11652	16.48416	34.97973	-1071.18	42.12364	162.8731
17.20618	15.5939	16.50766	16.30858	34.38369	-1070.97	41.77011	165.2408
17.18189	15.73214	16.55605	16.38978	34.14014	-1070.24	41.52949	166.0504
15.47359	17.10991	16.16342	16.63421	34.61887	-1069.96	41.74369	164.1005
17.09277	16.02917	16.63713	16.58281	33.65813	-1068.78	41.04934	167.6801
15.64346	17.23483	16.3168	16.75624	34.04867	-1068.47	41.21033	166.1247
17.38965	15.94463	16.77141	16.58384	33.31048	-1068.04	40.74899	168.9416
17.27399	16.1513	16.79302	16.6575	33.12419	-1067.38	40.54871	169.5818
17.52542	16.05459	16.89783	16.68824	32.83391	-1066.78	40.30218	170.6325
16.82739	16.88659	16.85861	16.95318	32.47423	-1065.06	39.84658	171.8896
16.28243	17.51429	16.81629	17.13712	32.24986	-1063.87	39.54379	172.6854
17.87344	16.30216	17.21051	16.94312	31.67076	-1063.75	39.2144	174.8329
17.60245	16.70222	17.23251	17.10758	31.35524	-1062.58	38.86665	176.0025
17.9757	16.57981	17.3946	17.14781	30.90207	-1061.62	38.47746	177.6771

16.82159	17.62687	17.18511	17.39494	30.97149	-1060.7	38.37258	177.4656
17.71729	17.06585	17.46059	17.37588	30.3804	-1059.87	37.92992	179.6732
17.85601	17.1027	17.56213	17.51038	29.96878	-1058.85	37.55347	181.2371
17.48665	17.62746	17.57226	17.6081	29.70555	-1057.67	37.23161	182.314
17.2881	17.86305	17.56593	17.74808	29.53485	-1057.02	37.04006	182.9858
17.76836	17.57173	17.71707	17.75105	29.1918	-1056.51	36.77859	184.3179
18.06004	17.57391	17.88993	17.81365	28.66248	-1055.24	36.30232	186.4493
18.55191	17.27293	18.03699	17.75817	28.38001	-1054.88	36.09723	187.5843
17.94711	17.86659	17.95469	17.94833	28.28329	-1054.03	35.9144	188.0366
18.70969	17.50544	18.23122	17.92412	27.62953	-1052.85	35.38686	190.7895
18.46274	17.9083	18.26941	18.11118	27.24837	-1051.52	34.98046	192.4871
18.77158	17.75767	18.38113	18.1182	26.97143	-1051.01	34.75756	193.7195
18.72003	17.96283	18.44485	18.27032	26.60198	-1049.92	34.39462	195.4192
18.82838	18.15303	18.58872	18.3496	26.08027	-1048.48	33.89881	197.9022
18.61253	18.44044	18.59229	18.45731	25.89742	-1047.75	33.68918	198.7978
19.03797	18.17326	18.72213	18.46619	25.60045	-1047.32	33.46957	200.2419
18.42121	18.84789	18.6584	18.63819	25.43431	-1046.22	33.21133	201.0965
18.93912	18.45465	18.78825	18.61305	25.20494	-1046.08	33.07308	202.2385
18.73518	18.64745	18.76306	18.78195	25.07237	-1045.57	32.92446	202.9195
19.20568	18.53465	18.99057	18.93638	24.33275	-1043.93	32.29119	206.7751
19.08149	18.89478	19.0666	18.88086	24.07627	-1042.94	32.00587	208.1675
19.05629	19.0576	19.12442	18.98855	23.77314	-1042.06	31.71335	209.8124
19.05629	19.0576	19.12442	18.98855	23.77314	-1042.06	31.71335	209.8124
19.57189	18.73114	19.2801	18.96005	23.45683	-1041.64	31.4909	211.5175
19.49572	18.98413	19.34881	19.11824	23.05311	-1040.42	31.09738	213.7538
19.75517	19.12834	19.56241	19.25947	22.29462	-1038.48	30.41638	217.9386
19.61111	19.35205	19.58177	19.34741	22.10767	-1037.8	30.21811	218.9849
19.63154	19.62215	19.70772	19.4906	21.54799	-1036.19	29.69283	222.0391
19.83214	19.49462	19.77782	19.62899	21.26645	-1035.66	29.47394	223.5523
19.93542	19.55014	19.86394	19.7245	20.92603	-1034.8	29.17522	225.3423
19.87146	19.77667	19.91809	19.65273	20.78105	-1034.22	29.01419	226.072
20.20762	19.55329	20.01551	19.63471	20.58887	-1034	28.88878	227.0089
20.01962	19.85192	20.04033	19.77364	20.3145	-1033.04	28.60782	228.4136
20.05715	19.95227	20.11768	20.05804	19.81489	-1031.75	28.17283	230.8451
20.31827	19.94537	20.26058	20.05924	19.41656	-1030.82	27.84206	232.6039
20.3486	19.96581	20.28736	20.08323	19.31502	-1030.56	27.75446	233.0437
20.77284	20.02703	20.55224	20.11139	18.5365	-1028.66	27.10307	236.0867
20.62252	20.25901	20.5764	20.32392	18.21817	-1027.67	26.80527	237.3158
20.71756	20.35108	20.66638	20.28741	17.97758	-1027.01	26.59451	238.0919
21.08551	20.31766	20.86155	20.35084	17.38444	-1025.65	26.12284	239.8415
21.07701	20.51483	20.94458	20.47377	16.98982	-1024.51	25.77574	240.9237
21.33962	20.37502	21.03472	20.47962	16.77102	-1024.15	25.62595	241.3965
20.91763	20.62624	20.92762	20.93031	16.59825	-1023.48	25.44942	242.024
20.97132	20.7466	21.01363	21.03028	16.23822	-1022.51	25.14751	242.7808
21.20434	20.97544	21.22564	20.81309	15.78149	-1021.2	24.75363	243.4527
21.70202	21.09033	21.56303	21.0111	14.63352	-1018.39	23.86058	244.888
21.95468	21.3218	21.81236	21.22289	13.68826	-1015.93	23.1219	245.6311
21.82735	21.67953	21.89773	21.40622	13.18917	-1014.39	22.70055	245.9582
22.09797	21.5326	21.99339	21.4482	12.92785	-1013.94	22.53703	246.066

22.30097	21.61436	22.14226	21.49412	12.44829	-1012.73	22.18243	246.2494
22.17595	21.96776	22.22683	21.67506	11.9544	-1011.21	21.77856	246.5128
22.3102	21.96203	22.30801	21.8065	11.61327	-1010.42	21.54395	246.6911
22.56245	21.97414	22.45491	21.7983	11.21019	-1009.47	21.26592	246.8428
22.31762	22.15583	22.42841	22.40973	10.68847	-1008.06	20.8881	247.4561
22.7708	22.47534	22.80396	22.19805	9.751853	-1005.55	20.21698	247.9578
22.85875	22.51626	22.88174	22.40149	9.341782	-1004.55	19.94746	248.4384
23.0327	22.54569	22.98684	22.37485	9.059939	-1003.86	19.76298	248.7035
23.09427	22.70928	23.09493	22.48977	8.611755	-1002.65	19.4553	249.2939
23.34185	22.78026	23.26715	22.55216	8.058587	-1001.29	19.10235	250.0531
23.22976	22.88138	23.24535	22.54934	8.094163	-1001.27	19.10771	250.0159
23.44995	22.96648	23.41121	22.67413	7.49824	-999.794	18.73349	250.9811
23.57443	23.094	23.53885	22.7762	7.016521	-998.542	18.42711	251.8518
23.50106	23.20676	23.54899	22.8411	6.902098	-998.159	18.3421	252.1091
23.60722	23.33995	23.67959	22.99212	6.381127	-996.811	18.01884	253.1721
23.67952	23.33761	23.71124	22.9702	6.301431	-996.628	17.97316	253.302
23.82364	23.47666	23.8569	23.09938	5.743412	-995.188	17.63458	254.4932
23.91906	23.51013	23.93063	23.24207	5.39812	-994.352	17.43732	255.3132
24.02851	23.7816	24.10719	23.32462	4.758091	-992.585	17.0433	256.7208
24.21998	23.81107	24.23433	23.43401	4.300613	-991.493	16.79355	257.7907
24.32953	23.93747	24.35533	23.55681	3.820855	-990.249	16.52114	258.9357
24.17103	23.82759	24.27789	24.50683	3.216772	-988.964	16.23591	261.2396
24.37257	24.10959	24.51465	24.64936	2.353955	-986.671	15.75891	263.2295
24.78875	24.39378	24.82141	23.92822	2.067842	-985.711	15.56552	263.001
24.54931	24.24026	24.68413	24.92414	1.602285	-984.793	15.37525	265.0748
24.59663	24.29102	24.73508	24.98478	1.392611	-984.254	15.26776	265.5608
24.97698	24.54994	25	24.07984	1.393241	-983.985	15.21713	264.5162
24.97698	24.54994	25	24.07984	1.393241	-983.985	15.21713	264.5162

Table S4. Validation of compositional spaces suggested by ML-MOGA framework with respect to direct MD simulations.

Fe	*Ni*	*Co*	*Cr*	*Al*	*MD_ ΔKE*	*ML_MOGA_ ΔKE*	*MD_ δ*	*ML_MOGA_ δ*	*MD_V_r*	*ML_MOGA__V_r*
16.90007	15.08533	16.10413	15.91047	36	1075.36	1073.73	43.30087	42.57	159.962	160.87
16.90007	15.08533	16.10413	15.91047	36	1075.36	1073.73	43.30087	42.57	159.962	160.87
16.61331	15.42387	16.0857	16.01528	35.86184	1074.679	1075.89	43.12512	44.25	160.2349	163.879
15.03718	16.8445	15.78988	16.32844	36	1073.538	1075.96	43.02711	42.85	159.3592	157.4365
15.81871	16.36196	16.03464	16.3057	35.479	1072.792	1070.72	42.63558	40.56	161.2098	159.685
24.97698	24.54994	25	24.07984	1.393241	983.9851	985.69	15.21713	13.89	264.5162	262.879
24.97698	24.54994	25	24.07984	1.393241	983.9851	985.69	15.21713	13.89	264.5162	262.879
24.59663	24.29102	24.73508	24.98478	1.392611	984.2541	983.26	15.26776	13.27	265.5608	264.87
24.54931	24.24026	24.68413	24.92414	1.602285	984.7928	982.23	15.37525	13.18	265.0748	264.89
24.78875	24.39378	24.82141	23.92822	2.067842	985.711	984.12	15.56552	13.25	263.001	262.4

References

1. Saha, S., Gupta, K.K., Maity, S.R. and Dey, S., 2022. Data-driven probabilistic performance of Wire EDM: A machine learning based approach. *Proceedings of the Institution of Mechanical Engineers, Part B: Journal of Engineering Manufacture*, *236*(6-7), pp.908-919.
2. Deb, K., 2011. *Multi-objective optimisation using evolutionary algorithms: an introduction* (pp. 3-34). Springer London.
